# Architecture, substructures, and dynamic assembly of STRIPAK complexes in Hippo signaling

**DOI:** 10.1038/s41421-018-0077-3

**Published:** 2019-01-08

**Authors:** Yang Tang, Min Chen, Li Zhou, Jian Ma, Yehua Li, Hui Zhang, Zhubing Shi, Qi Xu, Xiaoman Zhang, Ziyang Gao, Yun Zhao, Yunfeng Cheng, Shi Jiao, Zhaocai Zhou

**Affiliations:** 1 State Key Laboratory of Cell Biology, CAS Center for Excellence in Molecular Cell Science, Shanghai Institute of Biochemistry and Cell Biology, Chinese Academy of Sciences, University of Chinese Academy of Sciences, Shanghai, 200031 China; 2 The School of Life Science and Technology, ShanghaiTech University, Shanghai, 201210 China; 3 Department of Hematology and Institute of Clinical Science, Zhongshan Hospital, Fudan University, Shanghai, 200032 China

**Keywords:** X-ray crystallography, Molecular biology

## Abstract

Striatin-interacting phosphatases and kinases (STRIPAKs) are evolutionarily conserved supramolecular complexes, which have been implicated in the Hippo signaling pathway. Yet the topological structure and dynamic assembly of STRIPAK complexes remain elusive. Here, we report the overall architecture and substructures of a Hippo kinase-containing STRIPAK complex. PP2Aa/c-bound STRN3 directly contacts the Hippo kinase MST2 and also controls the loading of MST2 via two “arms” in a phosphorylation-dependent manner, one arm being STRIP1 and the other SIKE1-SLMAP. A decreased cell density triggered the dissociation of the STRIP1 arm from STRIPAK, reflecting the dynamic assembly of the complex upon sensing upstream signals. Crystallographic studies defined at atomic resolution the interface between STRN3 and SIKE1, and that between SIKE1 and SLMAP. Disrupting the complex assembly abrogated the regulatory effect of STRIPAK towards Hippo signaling. Collectively, our study revealed a “two-arm” assembly of STRIPAK with context-dependent dynamics, offering a framework for further studies on Hippo signaling and biological processes involving MST kinases.

## Introduction

Striatin-interacting phosphatase and kinase (STRIPAK) complexes are newly identified striatin (STRN)-mediated supramolecular assemblies that contain the protein phosphatase PP2A and a member of the germinal center kinase (GCK) family^1–3^ (Supplementary Fig. S1a). The mammalian STRN family of proteins includes STRN, STRN3 (also named SG2NA), and STRN4 (also named zinedin). Known kinase components in the STRIPAK complexes include GCKII subfamily members mammalian STE20-like protein kinase 1 (MST1, also named STK4) and MST2 (also named STK3); GCKIII subfamily members MST3 (also named STK24), MST4 (also named STK26), and STK25 (also named YSK1 or SOK1); GCKIV subfamily members misshapen-like kinase 1 (MINK1), TRAF2 and NCK-interacting protein kinase (TNIK), and mitogen-activated protein kinase (MAPK) kinase kinase kinase 4 (MAP4K4, also named HGK or NIK)^2,4–7^. As the PP2A regulatory B′′′ subunits, STRNs associate with the PP2A catalytic subunit (PP2Ac) via the PP2A scaffolding subunit (PP2Aa)^8–10^. Meanwhile, STRNs recruit GCK family members via different adaptor proteins such as cerebral cavernous malformations 3 (CCM3, also named PDCD10)^9^. Other major components of STRIPAK complexes include STRN-interacting protein 1 or 2 (STRIP1/2, also named FAM40A/B), MOB4 (also named phocein, MOB3, or MOBKL3), sarcolemmal membrane-associated protein (SLMAP), and its paralog tumor necrosis factor receptor-associated factor 3 (TRAF3)-interacting protein 3 (TRAF3IP3, also known as T3JAM), suppressor of IKBKE 1 (SIKE1) and its paralog fibroblast growth factor receptor 1 (FGFR1) oncogene partner 2 (FGFR1OP2), and cortactin-binding protein 2 (CTTNBP2) and its paralog CTTNBP2 N-terminal-like protein (CTTNBP2NL)^1,2^. It has been suggested that SLMAP/TRAF3IP3-SIKE1/FGFR1OP2 and CTTNBP2/CTTNBP2NL form mutually exclusive complexes with STRNs^2^.

STRIPAK complexes are highly conserved in eukaryotic organisms from fungi to mammals^11^. STRIPAK complexes as a whole, or as individual components, display multiple physiological functions and are associated with many pathological conditions^3,11^. Both PP2A and GCK kinases widely participate in growth, development, and immune responses, and a malfunction of these proteins frequently leads to diseases including cancers^12–15^. STRNs are implicated in the neuron development and nongenomic effects of nuclear receptors^16,17^. STRIP1/2 regulates cell morphology and migration via modulating cytoskeleton organization^18^. SLMAP could localize to different cellular compartments via its two different types of transmembrane domains^19^. An aberrant expression or mutation of SLMAP has been associated with type II diabetes, group I leiomyosarcoma, and Brugada syndrome^20–22^. TRAF3IP3 is involved in the development of T and B lymphocytes^23,24^, as well as in the function of regulatory T cells^25^. SIKE1 is an inhibitor of IKKε- and TBK1-mediated antiviral response, while FGFR1OP2 was reported to promote the closure of oral wounds^26,27^.

Among the kinase components of STRIPAK complex, MST1/2 are best known as upstream kinases of the mammalian Hippo signaling pathway^28,29^. In this pathway, MST1/2 activate downstream kinases LATS1/2, together with SAV1 and MOB1A/B. Then LATS1/2 phosphorylate the transcriptional coactivators YAP and TAZ to suppress their localization in the nucleus. Once MST1/2 kinases become inactive, unphosphorylated YAP/TAZ enter the nucleus, where they form complexes with transcription factors TEAD1–4 to regulate the expression of a large group of genes that usually promote cell proliferation and at the same time inhibit apoptosis. The expression of YAP/TAZ have been observed to be upregulated in many types of cancers^30^. In *Drosophila*, the knockdown of STRIPAK components promotes the activity and function of Hippo kinase (the homolog of human MST1/2)^4^. Recent studies have also shown that SLMAP most likely mediates the recruitment of MST1/2 into the STRIPAK complex^6,7,31,32^. GCKIII kinases MST3/4 can phosphorylate PPP1R14A-D to advance breast cancer metastasis via modulating the actomyosin cytoskeleton, while STRN3/4, STRIP1, and PP2A suppress the activity and function of MST3/4^33^. In *Caenorhabditis elegans*, multiple components of the STRIPAK complex including GCK-1 (homolog of GCKIII), CCM3, CASH-1 (homolog of STRNs), and FARL-11 (homolog of STRIP1/2) promote the extension of the canal by regulating endocytic recycling^34^.

STRIPAK complexes were identified several years ago, but their assembly and structures remain poorly understood, which impedes the deeper understanding of the functions of the STRIPAK complexes and, in particular, the regulatory mechanisms of Hippo signaling. Here, we used biochemical and crystallographic analyses to identify the direct interactions between the major components of the STRIPAK complex and pinpointed individual domains and specific amino acids required for complex assembly. Our results supported a “two-arm” architecture of the STRIPAK complex, with the PP2Aa/c-bound STRNs acting as an organizing center to hold Hippo or other MST kinases with STRIP1 as one arm and SIKE1-SLMAP as the other. Moreover, we determined the crystal structures of the second coiled-coil domain of SIKE1 alone, and in complex with an STRN3 peptide, as well as the structure of the first coiled-coil domain of SIKE1 in complex with the first coiled-coil domain of SLMAP. Disrupting STRIPAK complex assembly through domain deletions or structure-guided point mutations abolished its regulatory effects in Hippo signaling and gastric cancer cell proliferation. Furthermore, we found that the assembly of STRIPAK dynamically responds to cell density. A lower cell density readily triggers the dissociation of the STRIP1 arm, loosening the MST kinases from the vicinity of the PP2A core enzyme. Thus, our study indicated the activity of MST kinases to be intricately regulated through a “two-arm” system assembled in the STRIPAK complex in response to specific cellular cues such as cell density.

## Results

### STRN3 as an organizing center of STRIPAK

**Fig. 1:**
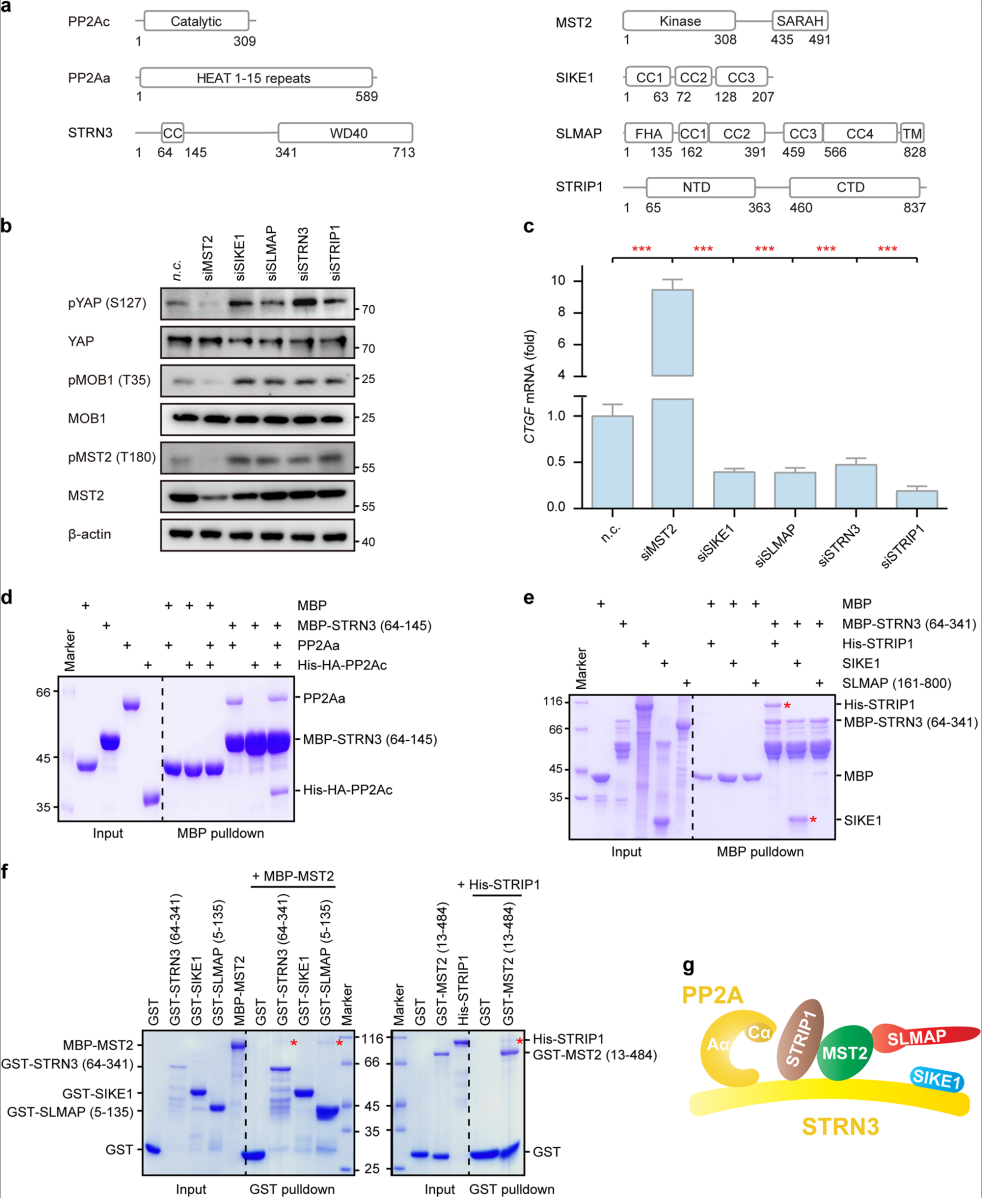
STRN3 as an organizing center of STRIPAK. **a** Schematic representation of the domain organization of the core STRIPAK components. CC, coiled-coil domain; WD40, WD40 repeat; Kinase, kinase domain; SARAH, Sav-RASSF-Hpo domain; NTD, N-terminal domain; CTD, C-terminal domain; FHA, forkhead-associated domain; TM, transmembrane domain. **b** Effect of the knockdown of STRIPAK components on the activation of Hippo signaling in HGC-27 cells. n.c., negative control (control siRNA). **c** Real-time PCR analysis of the expression levels of *CTGF* in HGC-27 cells transfected with the indicated siRNAs. Bar graphs represent the means ± SD. Experiments were repeated three times. Unpaired *t* tests were used to compare the difference between the two groups. *Significant relative to control, *p* < 0.05, **p < 0.01, ***p < 0.001. **d** MBP pulldown analysis of the PP2A holoenzyme containing STRN3 as its B′′′ subunit. The input and output samples were loaded on an SDS-PAGE gel followed by staining this gel with Coomassie brilliant blue (CBB). **e** MBP pulldown analysis of the interactions between core STRIPAK components and the scaffold protein STRN3. The input and output samples were loaded on an SDS-PAGE gel followed by staining this gel with CBB. **f** MBP pulldown analysis of the interactions between core STRIPAK components and the MST2 protein kinase. The input and output samples were loaded on an SDS-PAGE gel followed by staining this gel with CBB. **g** Model of the overall assembly of the STRIPAK complex

Previous studies have suggested that STRIPAK complexes contain some essential components including STRNs, PP2Aa/c, STRIP1/2, and GCKs, while the components SLMAP/TRAF3IP3 and CTTNBP2/CTTNBP2NL may form mutually exclusive complexes with STRN-associated STRIP1/2^2^. Also, SIKE1/FGFR1OP2 was indicated to interact with SLMAP/TRAF3IP3. In the present study, we chose the type of STRIPAK containing SLMAP and SIKE1 as a subject to have its assembly and structure analyzed. Recruitment of the mammalian Hippo kinase MST2 into the STRIPAK complex is thought to be important for the regulation of its kinase activity and, therefore, downstream signaling^3,4^. Here, we chose MST2 as one of the major kinase components of STRIPAK. Domain organizations of the major STRIPAK components studied in this work are shown in Fig. 1a.

We first examined in HGC-27 cells the regulatory effects of major STRIPAK components on Hippo signaling. As expected, the knockdown of MST2 markedly decreased the phosphorylation of its substrate MOB1 (T35), leading to an enhanced YAP activity as shown by the diminished YAP phosphorylation at S127 and the increased transcription of YAP target gene *CTGF* (Fig. 1b, c and Supplementary Fig. S1b). However, the depletion of the STRIPAK components SIKE1, SLMAP, STRN3, or STRIP1 all significantly promoted MST2 activation, resulting in elevated phosphorylation levels of MST2 (T180), MOB1 (T35), and YAP (S127), as well as a decreased expression of *CTGF* (Fig. 1b, c).

Consistent with our previous studies^35^, the coiled-coil domain (CC, amino acid residues 64–145) of STRN3 was shown using maltose-binding protein (MBP) pulldown assay to directly interact with the regulatory A subunit of PP2A (PP2Aa) but not with its catalytic C subunit (PP2Ac) (Fig. 1d). Therefore, the PP2Aa/c core subunits bind to STRN3 via PP2Aa to form a holoenzyme, confirming the robustness of our in vitro assay using purified recombinant proteins. For further analysis, we used an STRN3 fragment (residues 64–341) that is most likely responsible for the interaction with other STRIPAK components^9^. As shown in our pulldown assay, MBP-tagged STRN3, but not MBP itself, readily pulled down STRIP1 and SIKE1 but not the coiled-coil region (residues 161–800) of SLMAP, indicating direct interactions of STRN3 with STRIP1 and SIKE1, but not with SLMAP (Fig. 1e).

Subsequently, we analyzed the direct binding partners of the MST2 kinase within the STRIPAK complex. Our pulldown results revealed a direct interaction of MST2 with STRN3 and STRIP1, but not with SIKE1 (Fig. 1f). These results also showed the FHA domain (residues 5–135) of SLMAP to directly interact with MST2 (Fig. 1f), consistent with the previous studies^7,32,33^.

Taken together, these results indicated that PP2Aa/c-bound STRN3 acts as a central scaffold in the STRIPAK complex to directly contact not only MST2, but also STRIP1 and SIKE1, while MST2 directly binds to STRN3, STRIP1, and SLMAP (Fig. 1g).

### STRIP1 and SIKE1 as key adaptors of STRN3

**Fig. 2:**
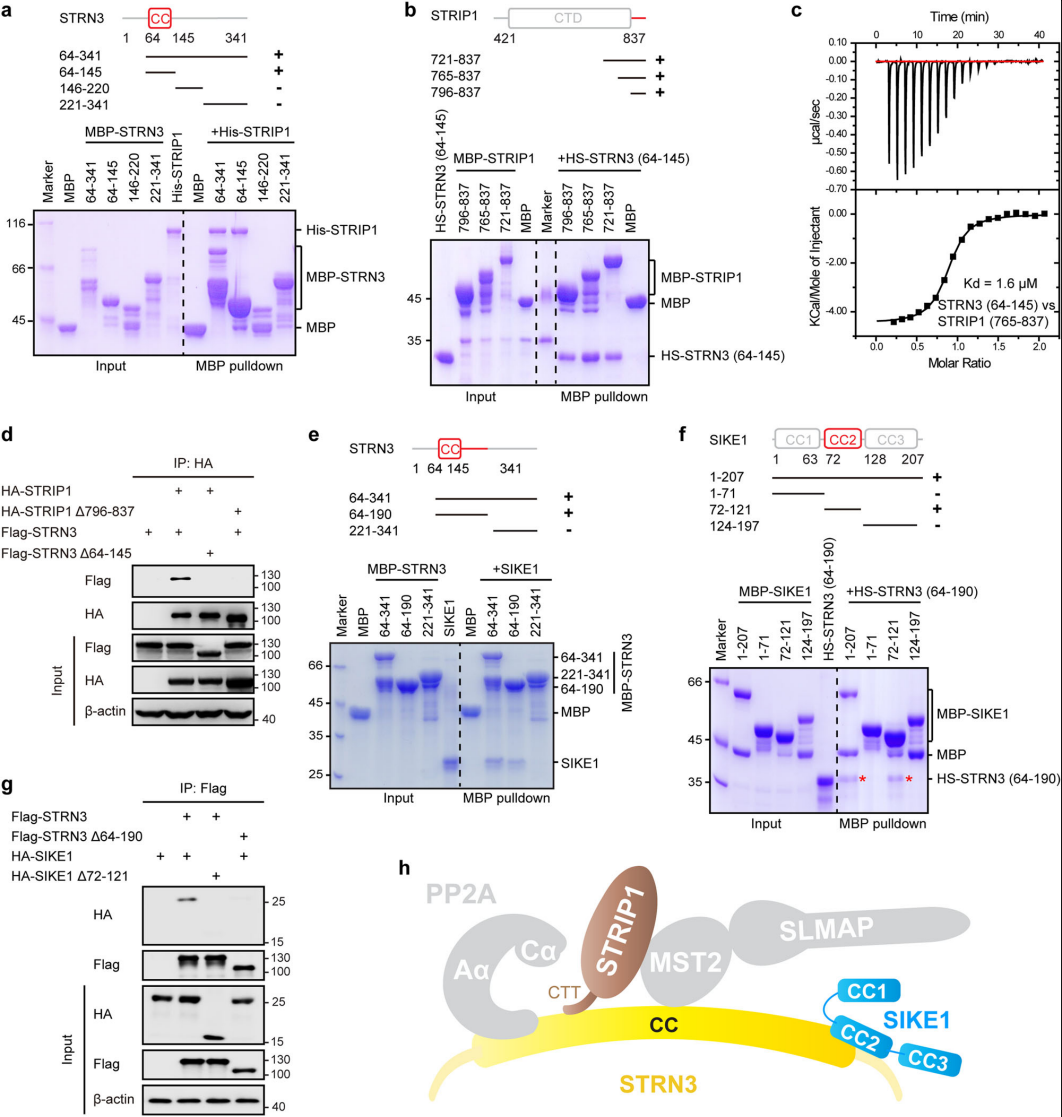
STRIP1 and SIKE1 as key adaptors of STRN3. **a** MBP-pulldown-based determination of the domains of STRN3 most responsible for binding STRIP1. The input and output samples were loaded on an SDS-PAGE gel followed by staining this gel with CBB. **b** MBP-pulldown-based determination of the STRN3-binding domains of STRIP1. The input and output samples were loaded on an SDS-PAGE gel followed by staining this gel with CBB. HS, His-SUMO tag. **c** ITC analysis of the interaction between STRN3 (64–145) and STRIP1 (765–837). **d** Co-immunoprecipitation and immunoblot analysis of STRN3 and STRIP1 in HEK293FT cells. Δ796–837, deletion of residues 796–837; Δ64–145, deletion of residues 64–145; IP, immunoprecipitation. **e** MBP-pulldown-based determination of the domains of STRN3 most responsible for binding SIKE1. The input and output samples were loaded on an SDS-PAGE gel followed by staining this gel with CBB. **f** MBP-pulldown-based determination of the STRN3-binding domain of SIKE1. The input and output samples were loaded on an SDS-PAGE gel followed by staining this gel with CBB. **g** Co-immunoprecipitation and immunoblot analysis of STRN3 and SIKE1 in HEK293FT cells. Δ64–190, deletion of residues 64–190; Δ72–121, deletion of residues 72–121. **h** Model of STRN3-STRIP1 and STRN3-SIKE1 assembly in the STRIPAK complex. CTT, C-terminal tail

To map the specific domains responsible for the interaction between STRN3 and STRIP1, we constructed different fragments of STRN3 and STRIP1. An MBP pulldown assay showed the coiled-coil domain of STRN3, but not the other fragments, to bind STRIP1 (Fig. 2a). On the other hand, the C-terminal domain (residues 421–837) of STRIP1, but not its N-terminal domain (residues 1–350 or 1–420), was pulled down by STRN3 coiled-coil domain (Supplementary Fig. S2a). Moreover, the C-terminal tail (CTT, residues 796–837) of STRIP1, but not the fragment containing residues 421–744, was sufficient for binding STRN3 (Fig. 2b and Supplementary Fig. S2b). Thus, we concluded the CTT of STRIP1 and coiled-coil domain of STRN3 to mediate the direct interaction between the two proteins. Isothermal titration calorimetry (ITC) was used to determine the equilibrium dissociation constant (Kd) of residues 765–837 of STRIP1 and residues 64–145 of STRN3 to be 1.6 μM (Fig. 2c). Consistent with these observations, deletion of the STRIP1 CTT region or STRN3 coiled-coil domain abolished the binding between these two proteins in cells (Fig. 2d).

Next, we mapped the specific regions required for STRN3 interaction with SIKE1. As shown in Fig. 2e, residues 64–190 of STRN3 but not other fragments could interact with SIKE1. Meanwhile, the second coiled-coil (CC2) region, namely residues 72–121 of SIKE1, was shown to bind STRN3 (Fig. 2f). Consistently, the deletion of STRN3 (64–190) or SIKE1 CC2 abolished the interaction between STRN3 and SIKE1 in cells (Fig. 2g). A further mapping experiment identified residues 166–190 of STRN3 as the minimal region for binding SIKE1 (Supplementary Fig. S2c). Moreover, ITC assay determined the Kd of residues 166–190 of STRN3 and SIKE1 CC2 to be 2.9 μM (Supplementary Fig. S2d).

Taken together, these results identified STRIP1 and SIKE1 as key adaptors of STRN3 that can directly bind to STRN3 through CTT of STRIP1 and the CC2 region of SIKE1, respectively (Fig. 2h).

### Structural basis of STRN3-SIKE1 assembly

**Fig. 3:**
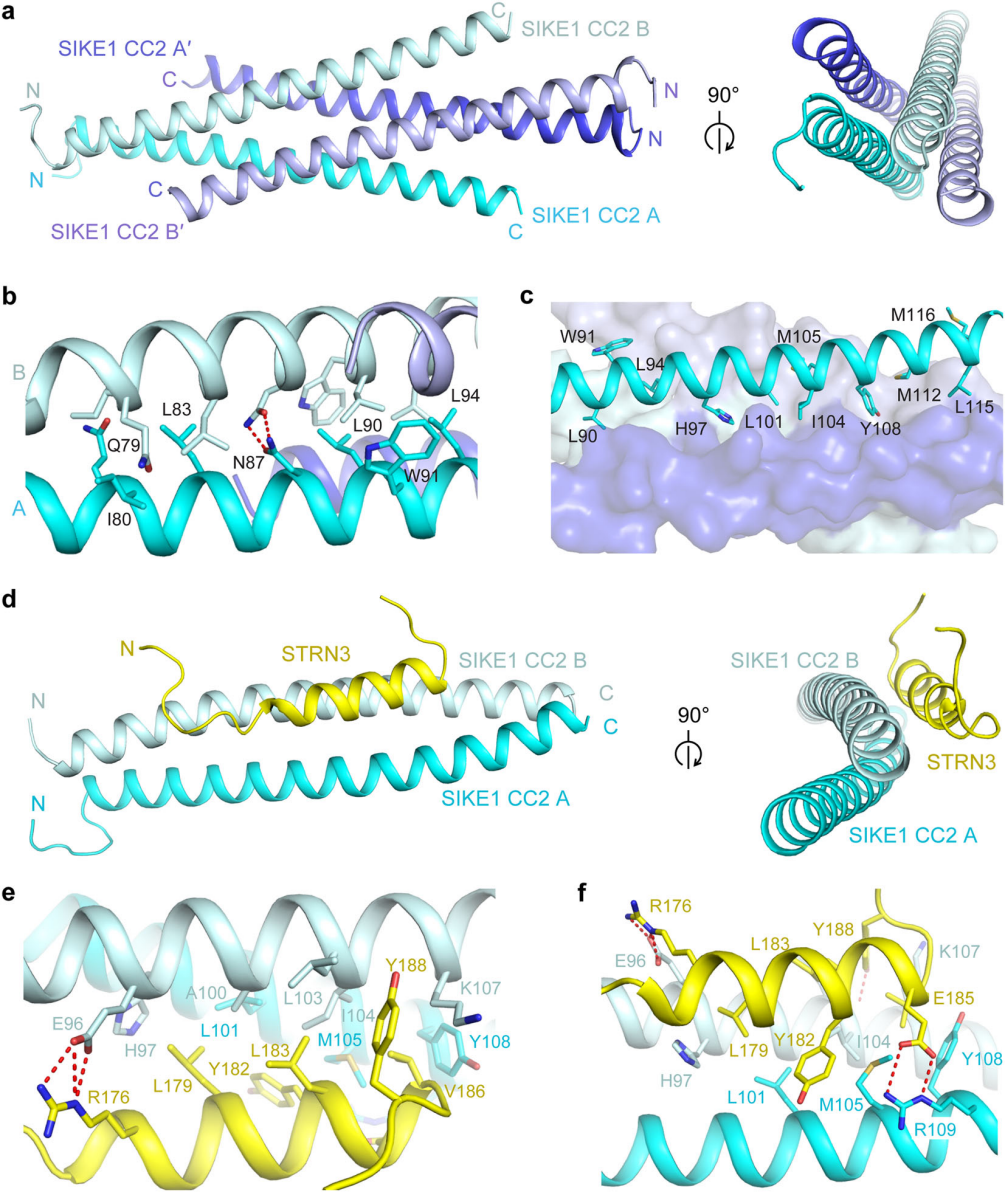
Structural basis for SIKE1 CC2 alone and SIKE1 CC2-STRN3 complex. **a** Cartoon views of the overall structure of apo SIKE1 CC2. Four chains of SIKE1 CC2 are colored cyan (chain A), palecyan (chain B), blue (chain A′), and lightblue (chain B′), respectively. **b** Interactions at the dimeric interface of SIKE1 CC2. **c** Interactions at the tetrameric interface of SIKE1 CC2. **d** Cartoon views of the overall structure of SIKE1 CC2-STRN3 complex. The two SIKE1 CC2 molecules are colored cyan (chain A) and palecyan (chain B), respectively, and the STRN3 peptides are colored yellow. **e,** **f** Zoomed-in views of SIKE1 and STRN3 interactions at the complex interface. The red dash lines represent hydrogen bonds or salt bridges. The interface residues are labeled and highlighted by the stick model

To further characterize the assembly of STRN3-SIKE1 complex at the atomic level, we solved the crystal structures of SIKE1 CC2 alone (apo state) and in complex with an STRN3 peptide (residues 166–190) at 1.5 and 1.75 Å resolution, respectively (Supplementary Tables S1 and S2). In the apo state, SIKE1 CC2 formed a symmetric homotetramer consisting of two parallel homodimers (chains A/B for one homodimer and A′/B′ for the other), namely a dimer of dimer with two dimeric units clamping each other in an antiparallel direction (Fig. 3a). As such, the N-terminal regions of SIKE1 CC2 formed a dimeric coiled coil, while the central and C-terminal regions formed a tetrameric coiled coil. Residues Q79, I80, L83, L90, W91, and L94 from each chain mediated CC2 homodimerization through hydrophobic interactions (Fig. 3b). In addition, N87 from chains A and B formed a pair of hydrogen bonds surrounding the axis of the coiled coil (Fig. 3b). Meanwhile, the four-helix bundle was stabilized by an extensive hydrophobic packing of residues L90, W91, L94, H97, L101, I104, M105, Y108, M112, L115, and M116 from each chain (Fig. 3c). Moreover, the coiled-coil assembly was further strengthened by hydrogen bonding among residues S93, L94, and Q98 from chain A, Y108 and R109 from chain B′, and Q111 from chain A′, as well as salt bridges formed between residues E102 from chain A and R109 from chain B′ (Supplementary Fig. S3).

In the structure of the STRN3-SIKE1 CC2 complex, two SIKE1 CC2 molecules (chains A and B) formed a homodimer to bind one STRN3 peptide, resulting in a 2:1 heterotrimer (Fig. 3d). STRN3 folded as an alpha helix with a C-terminal turn. Many residues mediating homotetramerization of SIKE1 CC2 in the apo state were now found to contact the STRN3 peptide. For example, residues H97, A100, L103, I104, and K107 in chain B, and L101, M105, and Y108 in chain A of SIKE1 CC2 formed a hydrophobic patch with residues L179, Y182, L183, V186, and Y188 of STRN3 (Fig. 3e, f). In addition, the side chains of residues E96 in chain B and R109 in chain A of SIKE1 CC2 formed two salt bridges and three hydrogen bonds with R176 and E185 of STRN3 (Fig. 3e, f).

**Fig. 4:**
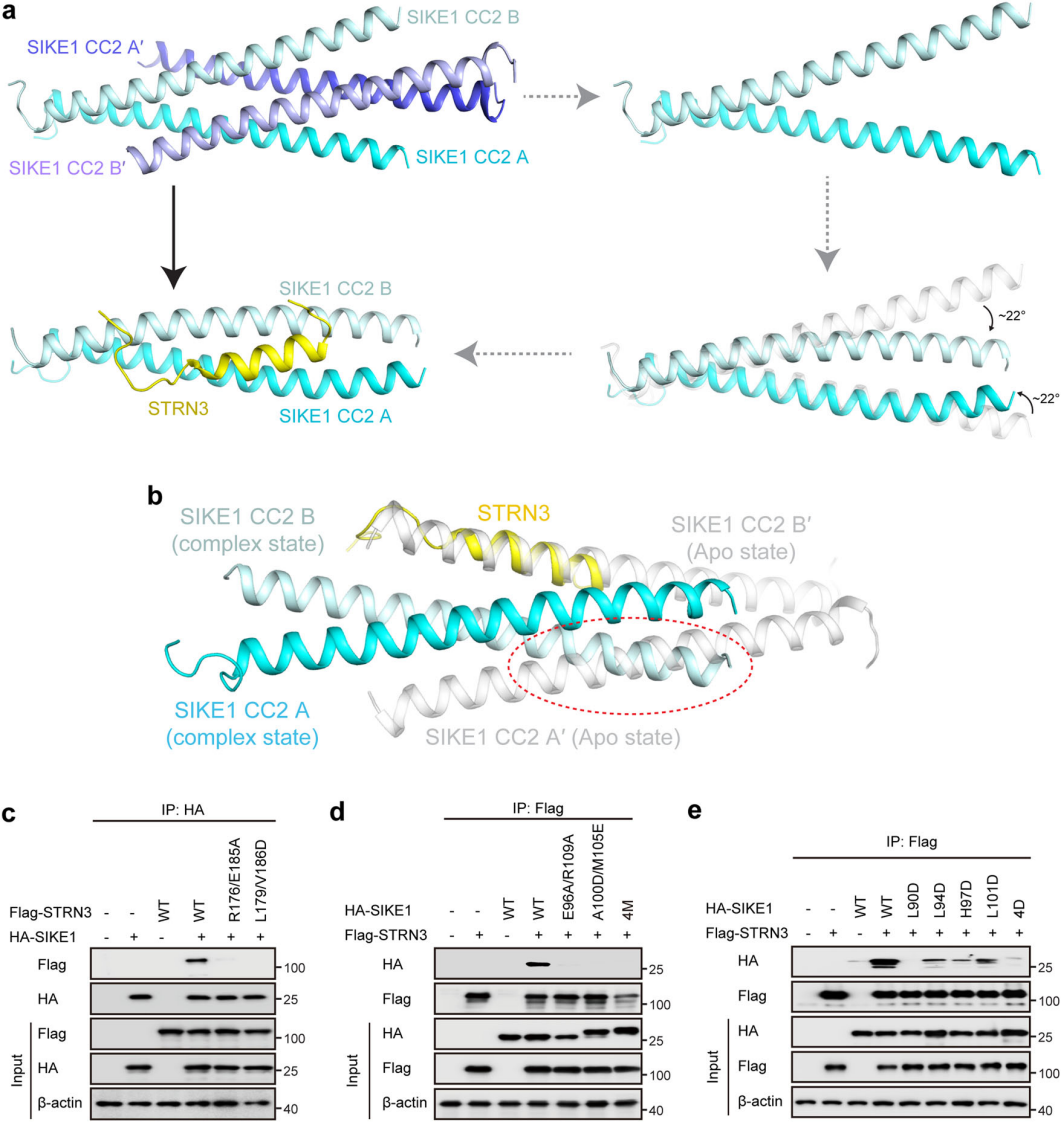
Assembly of SIKE1 CC2-STRN3 complex. **a** Overall structural comparison of apo SIKE1 CC2 with SIKE1 CC2-STRN3 complex. **b** Structure-based explanation of the 2:1 stoichiometry in SIKE1 CC2-STRN3 complex. **c**–**e** Co-immunoprecipitation and immunoblot analysis of the interactions between wildtype or mutant forms of SIKE1 and STRN3 in lysates of HEK293FT cells transfected with the indicated plasmids. 4M, E96A/R109A/A100D/M105E; 4D, L90D/L94D/H97D/L101D

Structural comparison revealed significant conformational changes in SIKE1 CC2 upon STRN3 binding. As a first step, the SIKE1 CC2 homotetramer observed in the apo state would have to disassemble, producing two homodimers with an exposed binding site for STRN3 (Fig. 4a). Secondly, in the presence of STRN3, SIKE1 CC2 became an asymmetric homodimer (chains A and B) with C-terminal regions in both chains bending closer to each other (about 22°, Fig. 4a). The bended chain B occupied a position corresponding to chain A′ of SIKE1 CC2 in the apo state (Fig. 4a, b). Since the STRN3 bound to SIKE1 as if it were a helical chain of SIKE1, the bending of SIKE1 CC2 thus blocked the binding of another STRN3 peptide, explaining the 2:1 stoichiometry (Fig. 4a, b).

Structure-guided mutational analysis showed that mutations of STRN3 (R176A/E185A and L179D/V186D) or SIKE1 (E96A/R109A and A100D/M105E) on the SIKE1-STRN3 interface disrupted the complex formation (Fig. 4c, d). Notably, mutations on the SIKE1 dimerization interface (L90D, L94D, H97D, L101D) also impaired the complex assembly (Fig. 4e). These results identified the structural features of the STRN3-SIKE1 complex assembly, and also highlighted the importance of SIKE1 homodimerization for binding STRN3.

### STRN3 directly interacts with MST2

**Fig. 5:**
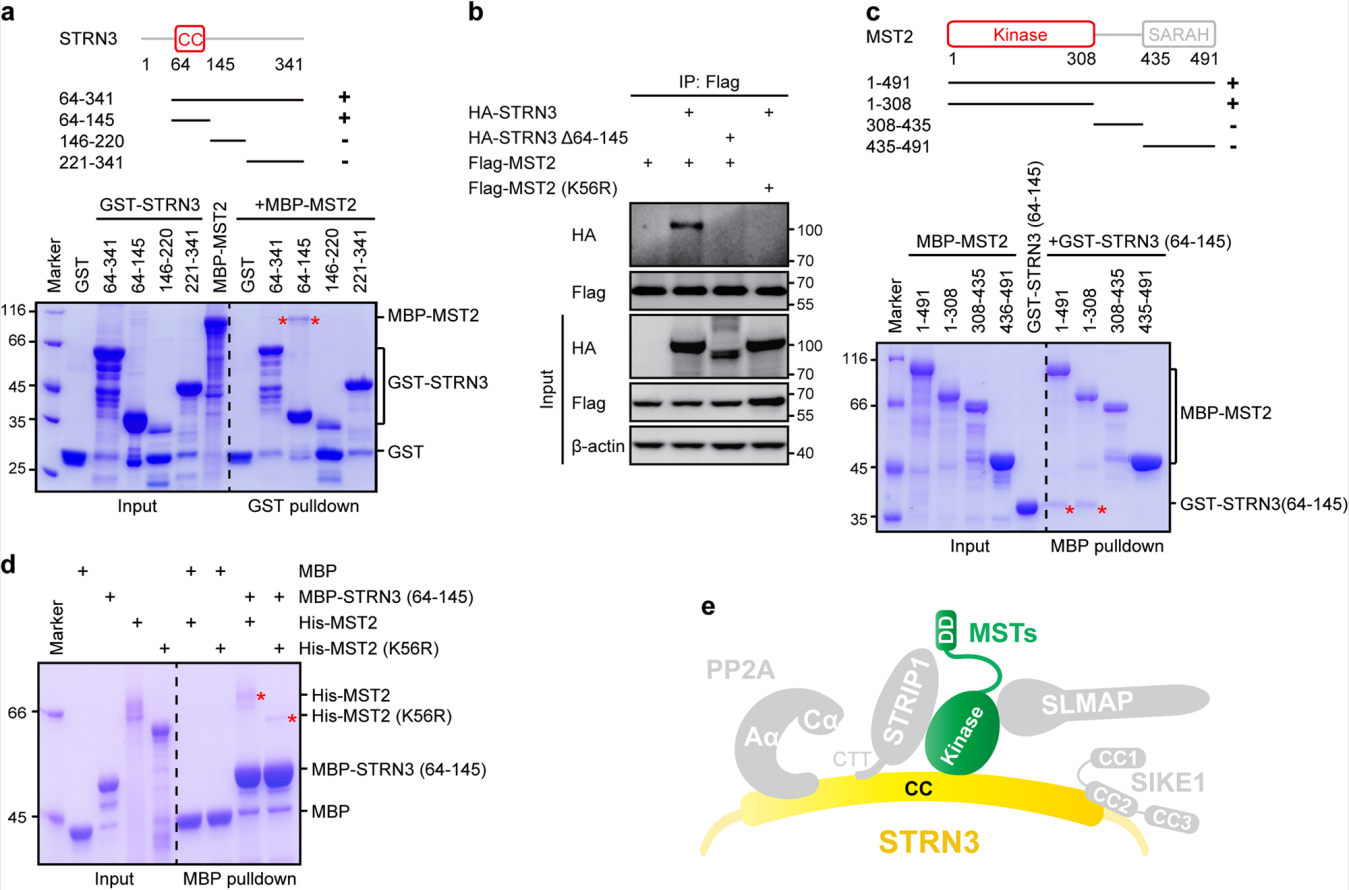
STRN3 directly interacts with MST2. **a** GST-pulldown-based determination of the MST2-binding domain of STRN3. The input and output samples were loaded on an SDS-PAGE gel followed by staining this gel with CBB. **b** Co-immunoprecipitation and immunoblot analysis of STRN3 and MST2 in HEK293FT cells. Δ64–145, deletion of residues 64–145; K56R, a form of MST2 displaying no kinase activity. **c** MBP-pulldown-based determination of the STRN3-binding domain of MST2. The input and output samples were loaded on an SDS-PAGE gel followed by staining this gel with CBB. **d** MBP pulldown analysis of the interaction between STRN3 and the wildtype or mutant form of MST2. The input and output samples were loaded on an SDS-PAGE gel followed by staining this gel with CBB. **e** Model of STRN3-MST2 assembly in the STRIPAK complex. Kinase, kinase domain; DD, dimerization domain, which is the SARAH domain in MST2 and DD in MST4

Given the ability of STRN3 to directly interact with MST2 (Fig. 1f), we then mapped which part of STRN3 mediates its association with MST2. Our pulldown results showed the STRN3 coiled-coil domain, but not other fragments including residues 146–220 or 221–341, to bind MST2 (Fig. 5a). Notably, a longer fragment (residues 64–341) of STRN3 showed a significantly reduced ability to bind MST2 than did the coiled-coil domain of STRN3 (Fig. 5a), suggesting the inhibitory role of the fragment containing residues 146–341. A subsequently performed co-immunoprecipitation assay confirmed an association of wildtype STRN3, but not of its coiled-coil domain deletion form (Δ64–145), with MST2 in cells (Fig. 5b). Further domain mapping revealed that the kinase domain of MST2 was responsible for STRN3 binding (Fig. 5c). Similarly, MST4 was also determined to bind to the coiled-coil domain of STRN3 through its N-terminal kinase domain (Supplementary Fig. S4a).

Given that MST2 kinase binds to multiple adaptors or regulators in a phosphorylation-dependent manner^36^, we next tested whether STRN3-MST2 association depends on MST2 phosphorylation. When treated with λ-protein phosphatase (λPPase), MST2 still bound STRN3, although to a lesser extent (Supplementary Fig. S4b). Meanwhile, the mutation of K56 of MST2 to R, which abrogates the kinase activity of MST2^37^, yielded a significantly reduced interaction between MST2 and STRN3 (Fig. 5d). Moreover, the kinase-inactive form of MST2 (K56R) failed to bind STRN3 in cells (Fig. 5b). Taken together, these results indicated that the coiled-coil domain of STRN3 directly interacts with the kinase domain of MST2 possibly in a manner partially dependent on MST2 autophosphorylation (Fig. 5e).

### STRIP1 and SLMAP bind MSTs in a phosphorylation-dependent manner

**Fig. 6:**
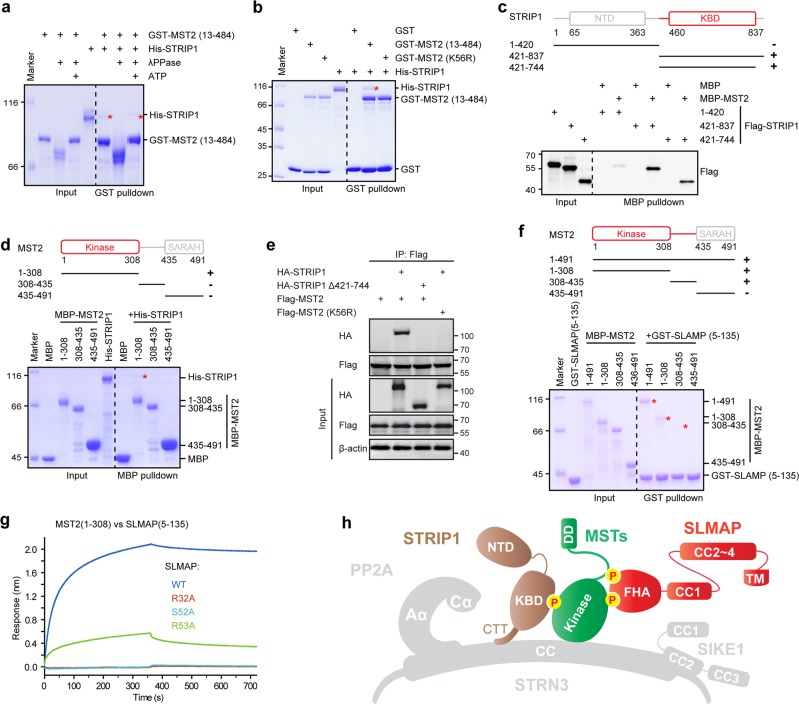
STRIP1 and SLMAP bind MSTs in a phosphorylation-dependent manner. **a** Pulldown analysis of the interaction between MST2/pMST2 and STRIP1. Purified protein of GST-tagged MST2 was first dephosphorylated using lambda-phosphatase (λPPase) or further autophosphorylated in the presence of ATP before use. The input and output samples were loaded on an SDS-PAGE gel followed by staining this gel with CBB. **b** MBP pulldown analysis of the interaction between STRIP1 and the wildtype or kinase-inactive mutant form of MST2. The input and output samples were loaded on an SDS-PAGE gel followed by staining this gel with CBB. **c** MBP-pulldown-based determination of the MST2-binding domain of STRIP1. Fragments of Flag-STRIP1 were obtained using an in vitro cell-free system. The input and output samples were loaded on an SDS-PAGE gel followed by immunoblot analysis. **d** MBP-pulldown-based determination of the STRIP1-binding domain of MST2. The input and output samples were loaded on an SDS-PAGE gel followed by staining this gel with CBB. **e** Co-immunoprecipitation and immunoblot analysis of MST2 and STRIP1 in HEK293FT cells. Δ421–744, deletion of residues 421–744; K56R, a kinase-inactive form of MST2. **f** GST-pulldown-based determination of the SLMAP-binding domain of MST2. The input and output samples were loaded on an SDS-PAGE gel followed by staining this gel with CBB. **g** Bio-layer interferometry (BLI) analysis of the interactions between the MST2 kinase domain and the indicated SLMAP (FHA) mutants. **h** Model of STRIP1-MST2-SLMAP assembly in the STRIPAK complex. NTD, N-terminal domain; KBD, kinase-binding domain

To further investigate how kinase components are recruited into STRIPAK, we analyzed the interaction of STRIP1 with GCK members including MST2, MST4, and OSR1. Our glutathione S-transferase (GST) pulldown assay showed a direct interaction of STRIP1 with MST2 and MST4, but not with OSR1 (Supplementary Fig. S5a). Considering that both MST2 and MST4 can undergo autophosphorylation whereas OSR1 is constitutively inactive in the absence of its upstream kinase WNK^15,38^, we reasoned that STRIP1 may associate with STRIPAK kinases in a phosphorylation-dependent manner. In keeping with this notion, MST2 appeared to have lost its ability to bind STRIP1 when treated with λPPase but had its interaction with STRIP1 restored once treated with adenosine triphosphate and magnesium ion (Fig. 6a). Moreover, the kinase-inactive form of MST2 (K56R) did not bind STRIP1 (Fig. 6b). These results indicated that STRIP1 helps to recruit the STRIPAK kinases in a phosphorylation-dependent manner.

Subsequently, we mapped the specific region responsible for the interaction between MST2/4 and STRIP1. Using STRIP1 expressed in an in vitro cell-free system, we found an association of MST kinases with the C-terminal region (residues 421–837) of STRIP1, but not with its N-terminal domain (NTD, residues 1–420) (Fig. 6c and Supplementary Fig. S5b). Further mapping revealed that a fragment of STRIP1 containing residues 421–744 was sufficient to bind the MST2 kinase (Fig. 6c), while the C-terminal tail region containing residues 721–837, essential for STRN3 binding, failed to pull down the kinases (Supplementary Fig. S5c). Based on these results, we concluded residues 421–744 of STRIP1 to be its kinase-binding domain (KBD). Further pulldown assays showed the kinase domains of MST2/4, namely MST2 residues 1–308 and MST4 residues 1–300, but not other regions, to be necessary and sufficient for binding STRIP1 (Fig. 6d and Supplementary Fig. S5d). Consistent with our pulldown analysis, either the deletion of STRIP1 KBD (Δ421–744) or the mutation of K56 of MST2 to R disrupted the association between these two proteins in HEK293FT cells (Fig. 6e).

The previous co-immunoprecipitation assay suggested that SLMAP associates through its FHA domain with MST1/2, with this association dependent upon MST1/2 being activated^7^. To dissect how such an association contributes to the recruitment of kinases into STRIPAK complexes, we examined the direct interaction of SLMAP with GCK kinases. Indeed, our results showed a direct interaction between the FHA domain (amino acid residues 5–135) of SLMAP and MST2 (Supplementary Fig. S6a). Moreover, SLMAP was also observed to bind to MST4, but not to OSR1 (Supplementary Fig. S6a), suggesting that SLMAP recognizes phosphorylated GCKs. Supporting this notion, MST2 or MST4 treated with λPPase did not bind SLMAP, while a subsequent treatment with adenosine triphosphate to induce its autophosphorylation restored MST2-SLMAP association (Supplementary Fig. S6b and S6c). Furthermore, the MST2 K56R mutant did not interact with SLMAP (Supplementary Fig. S6d), confirming that MST2 interacts with SLMAP in a phosphorylation-dependent manner. Meanwhile, our pulldown assay showed an interaction of MST2 with the FHA domain but with no other region of SLMAP (Supplementary Fig. S6e). Deletion of the SLMAP FHA domain or mutation of K56 to R in MST2 disrupted the association between these two proteins in HEK293FT cells (Supplementary Fig. S6f). On the other hand, consistent with a recent crystallographic study^32^, we found the middle linker region (residues 308–435) of MST2 to associate with SLMAP in a phosphorylation-dependent manner (Fig. 6f). However, in addition to this linker region, our results also indicated the kinase domain of MST2 to strongly bind SLMAP (Fig. 6f). More importantly, in the case of MST4, its kinase domain was determined to be solely responsible for binding SLMAP (Supplementary Fig. S6g). Together, these results indicated that the FHA domain of SLMAP recognizes the kinase domain of MST2 and that of MST4, as well as the linker region of MST2.

Recently, the crystal structure of SLMAP FHA domain in complex with the middle linker region of MST2 was reported^32^, which identified residues R32, S52, and R53 of SLMAP as a docking site for kinases. Notably, while wildtype SLMAP was shown, as described above, to strongly bind the kinase domain of MST2, this interaction was nearly abolished by a mutation of the docking site, in particular by a mutation of R32 to A, as well as by a mutation of S52 to A (Fig. 6g and Supplementary Fig. S6h).

Taken together, these results not only identified residues 421–744 of STRIP1 as a KBD that can interact with the kinase domain of MST2/4 (Fig. 6h) but also revealed that the FHA domain of SLMAP directly interacts in a conserved manner with the phosphorylated kinase domains of MST2 and MST4, and with the phosphorylated linker region of MST2 (Fig. 6h).

### SIKE1 recruits SLMAP to STRN3

**Fig. 7:**
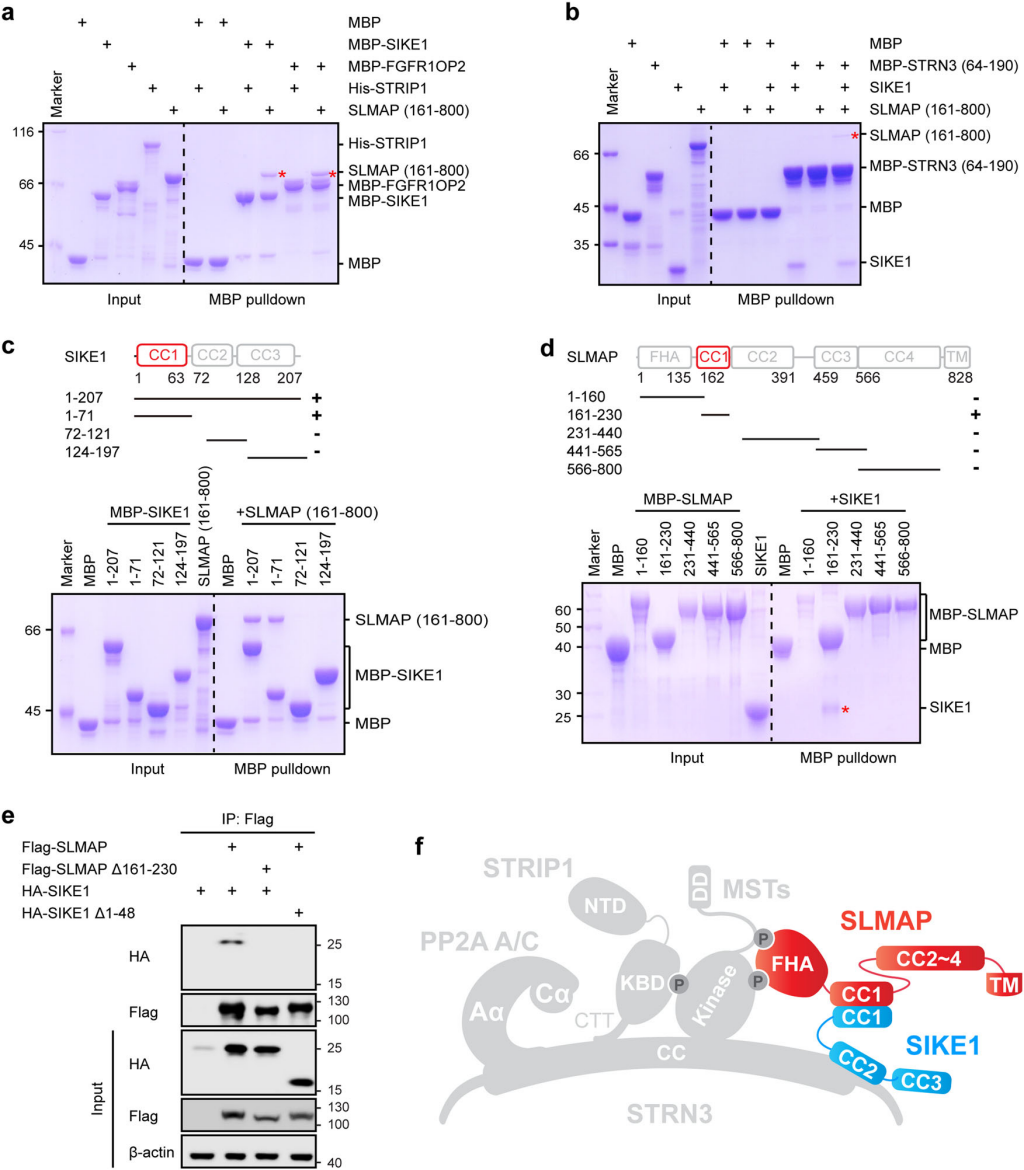
SIKE1 recruits SLMAP to STRN3. **a** MBP pulldown analysis of the interaction between SIKE1/FGFR1OP2 and SLMAP or STRIP1. FGFR1OP2, a homolog of SIKE1. The input and output samples were loaded on an SDS-PAGE gel followed by staining this gel with CBB. **b** MBP pulldown analysis of the interaction between SLMAP and STRN3 in the absence or presence of SIKE1. The input and output samples were loaded on an SDS-PAGE gel followed by staining this gel with CBB. **c** MBP-pulldown-based determination of the SLMAP-binding domain of SIKE1. The input and output samples were loaded on an SDS-PAGE gel followed by staining this gel with CBB. **d** MBP-pulldown-based determination of the SIKE1-binding domain of SLMAP. The input and output samples were loaded on an SDS-PAGE gel followed by staining this gel with CBB. **e** Co-immunoprecipitation and immunoblot analysis of SLMAP and SIKE1 in HEK293FT cells. Δ161–230, deletion of residues 161–230; Δ1–48, deletion of residues 1–48. **f** Model of SLMAP-SIKE1 assembly in the STRIPAK complex

Given the presence of coiled-coil domains in both SIKE1 and SLMAP, with such domains usually mediating homo- or heterodimerization^39^, and given the lack of direct binding of SLMAP to STRN3, determined above to serve as the central scaffold in the STRIPAK complex, we suspected that SLMAP may be recruited to STRN3 via SIKE1. To test this possibility, we purified proteins of SLMAP/TRAF3IP3 and SIKE1/FGFR1OP2 and analyzed the interactions between these components using in vitro pulldown assays. Indeed, MBP-tagged SIKE1, as well as its paralog FGFR1OP2, was observed to readily pulldown SLMAP, but not STRIP1 (Fig. 7a). Moreover, the coiled-coil regions of SLMAP (CC1-2, residues 161–565) and TRAF3IP3 (residues 188–513) were shown to mediate direct interactions with SIKE1, but not with STRIP1 (Supplementary Fig. S7a). More importantly, SLMAP associated with STRN3 only in the presence of SIKE1 (Fig. 7b). Together, these results indicated that SLMAP is recruited to STRN3 via SIKE1.

Next, we mapped the specific regions important for the interaction between SIKE1 and SLMAP. An MBP pulldown assay showed that SIKE1, through its N-terminal coiled-coil domain (CC1, residues 1–71), directly interacted with the coiled-coil regions of SLMAP (Fig. 7c). On the other hand, only the first coiled-coil (CC1) of SLMAP captured SIKE1 (Fig. 7d). These results indicated SIKE1 and SLMAP to interact with each other through their respective coiled-coil domains. Deletion of either SIKE1 CC1 or SLMAP CC1 disrupted the association between these proteins in HEK293FT cells (Fig. 7e). A further mapping experiment identified residues 5–65 of SIKE1 and residues 167–226 of SLMAP as the minimal regions mediating the interactions between the two proteins with a Kd of 6.9 μM (Supplementary Fig. S7b and S7c). Moreover, the minimal binding domains of SIKE1 and SLMAP formed a stable complex as shown by the gel filtration assay (Supplementary Fig. S7d).

Since both PP2Aa and STRIP1 were shown, as described above, to bind the coiled-coil domain of STRN3, we next tested whether STRN3 can recruit both PP2A and STRIP1 at the same time. As expected, STRIP1 could associate with PP2Aa in the presence of STRN3 (Supplementary Fig. S7e). Taken together, these data indicated that STRN3 can recruit STRIP1, SIKE1, and MST2 directly and SLMAP indirectly through SIKE1 (Fig. 7f).

### Structural basis of SIKE1-SLMAP assembly

**Fig. 8:**
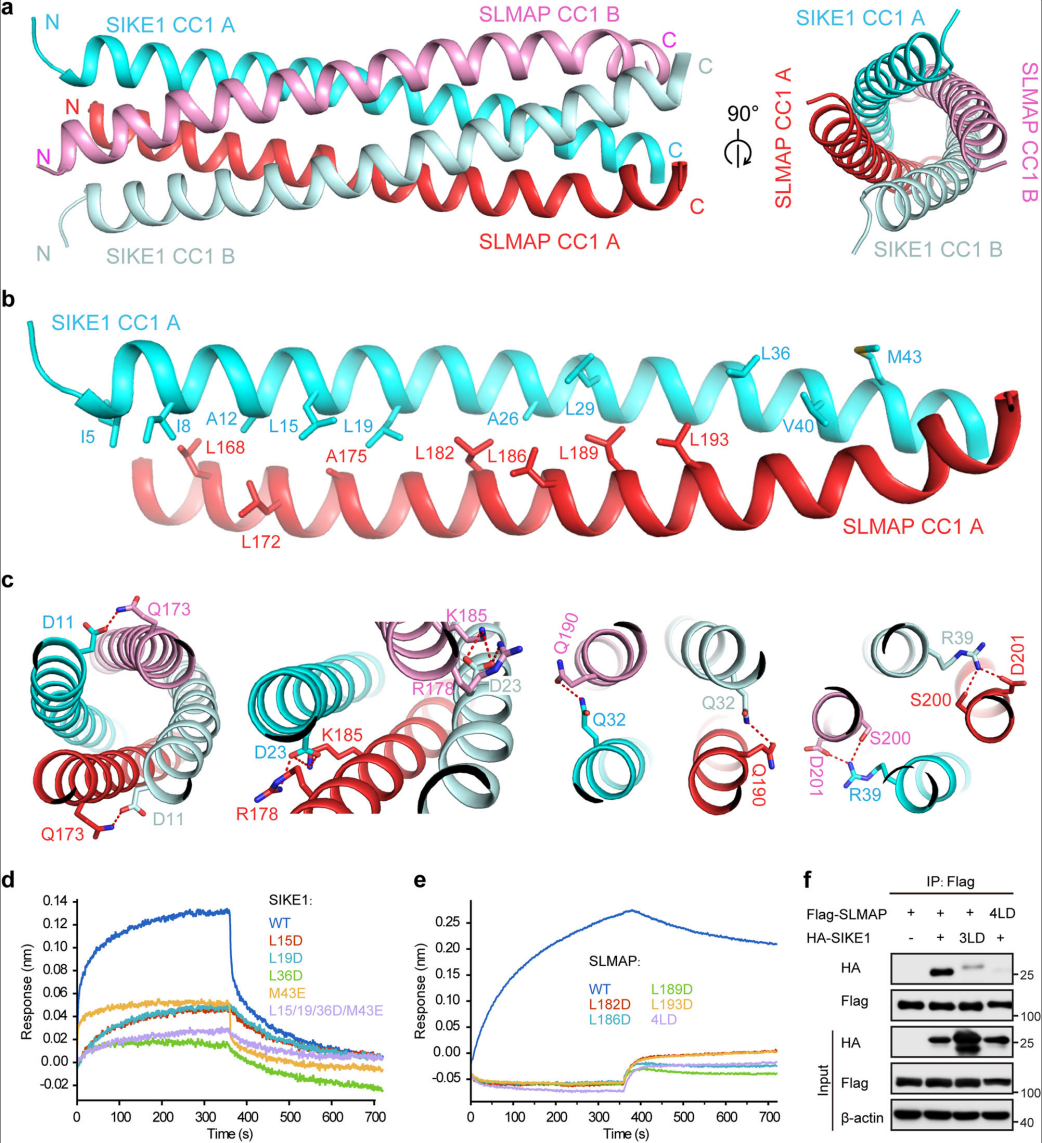
Structural basis of SIKE1-SLMAP assembly. **a** Cartoon views of the overall structure of SIKE1 CC1-SLMAP CC1 complex. Two structurally symmetrical SIKE1 CC1-SLMAP CC1 dimers (SIKE1 CC1 A-SLMAP CC1 A and SIKE1 CC1 B-SLMAP CC1 B) patch together to form a tetramer. Two chains of SIKE1 CC1 are colored cyan (chain A) and palecyan (chain B); and two SLMAP CC1 molecules are colored red (chain A) and pink (chain B). **b** Hydrophobic interactions at the interface of SIKE1 CC1-SLAMP CC1 complex. **c** Polar interactions at the interface of SIKE1 CC1-SLAMP CC1 complex. **d** BLI analysis of interactions of wildtype or mutant SIKE1 with wildtype SLMAP. Colored curves are the experimental traces of BLI experiments for mutations in SIKE1. **e** BLI analysis of the interactions of wildtype or mutant SLMAP with wildtype SIKE1. Colored curves are the experimental traces of BLI experiments for mutations in SLMAP. 4LD, L182D/L186D/L189D/L193D. **f** Co-immunoprecipitation and immunoblot analysis of the interactions between wildtype or mutant forms of SIKE1 and SLMAP in lysates of HEK293FT cells transfected with the indicated plasmids. 3LD, L15D/L19D/L36D. The red dash lines represent hydrogen bonds or salt bridges. The interface residues are labeled and highlighted by stick model

To further dissect at atomic level the SIKE1-SLMAP assembly, we determined the crystal structure of SIKE1 CC1 (residues 5–65) in complex with SLMAP CC1 (residues 167–226) at 2.3 Å resolution (Supplementary Table S3). As shown in Fig. 8a, an SIKE1 CC1 homodimer was interlaced with an SLMAP CC1 homodimer to assemble as a parallel four-helical bundle. A group of hydrophobic residues including I5, I8, A12, L15, L19, A26, L29, L36, V40, and M43 from SIKE1 CC1, and L168, L172, A175, L182, L186, L189, and L193 from SLMAP CC1 packed together to form a hydrophobic core (Fig. 8b). Moreover, residues D11, D23, Q32, and R39 from SIKE1 CC1 contacted residues Q173, R178, K185, Q190, S200, and D201 from SLMAP CC1 through hydrogen bonds and salt bridges (Fig. 8c).

Subsequently, we performed structure-guided mutational analysis. As shown in the bio-layer interferometry and MBP pulldown results, except for SIKE1-M43E, all other mutations on the complex interface including SIKE1 (L15D, L19D, L36D) and SLMAP (L182D, L186D, L189D, L193D) abolished the interactions between SIKE1 and SLMAP (Fig. 8d, e and Supplementary Fig. S8a, S8b). These observations were further confirmed by the co-immunoprecipitation assay showing that the mutant forms of SIKE1 and SLMAP were disabled for complex formation (Fig. 8f). Taken together, these results revealed a 2:2 heterotetrameric assembly of SIKE1-SLMAP and identified the leucine residues on the complex interface, namely L15, L19, and L36 of SIKE1 and L182, L186, L189, and L193 of SLMAP essential for SIKE1-SLMAP assembly.

### STRIPAK regulates Hippo signaling dependent on complex assembly

**Fig. 9:**
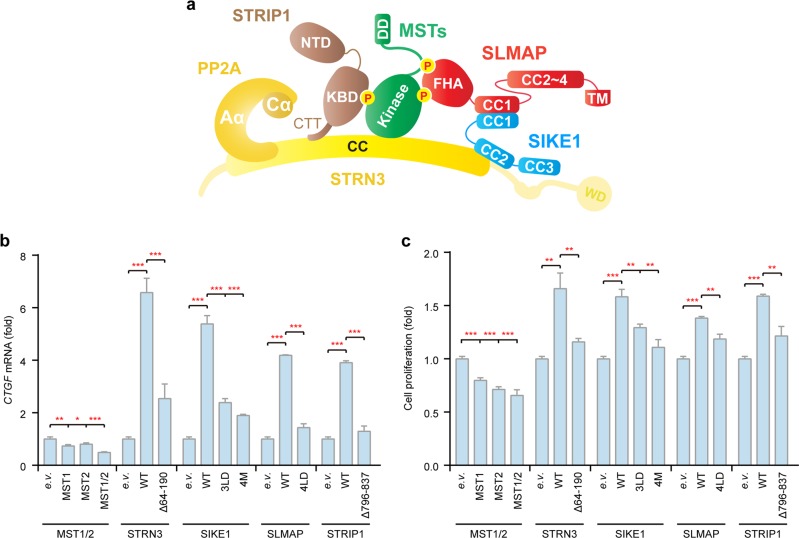
STRIPAK regulates Hippo signaling dependent on complex assembly. **a** Proposed model of the topological structure of the STRIPAK complex. **b** Real-time PCR analysis of expression levels of *CTGF* in HGC-27 cells transfected with the indicated plasmids. e.v., empty vector; WT, wildtype; Δ64–190, deletion of residues 64–190; Δ796–837, deletion of residues 796–837; 3LD, L15D/L19D/L36D; 4M, E96A/R109A/A100D/M105E; 4LD, L182D/L186D/L189D/L193D. **c** Cell proliferation rate of HGC-27 cells transfected with the indicated plasmids (day 3). Bar graphs represent the means ± SD. Experiments were repeated three times. Unpaired *t* tests were used to compare the difference between the two groups. *Significant relative to the control or the wildtype group, *p* < 0.05, **p < 0.01, ***p < 0.001. n.s., no statistical significance

Our biochemical and structural analysis pointed to a “two-arm” model for STRIPAK complex assembly, with PP2Aa/c-bound STRN3 acting as an organizing center to hold the Hippo/MST kinases with STRIP1 as one arm and SIKE1-SLMAP as the other (Fig. 9a). To further verify this model, we examined in HGC-27 cells the regulatory effects of STRN3 and its two “arms” on Hippo signaling. In contrast to the suppressive effect of MST1/2 on YAP target gene’s expression, the overexpression of STRN3 or its two “arms” markedly enhanced the expression of *CTGF* (Fig. 9b). However, the mutants of STRN3 or its two “arms”, which disrupted the assembly of the STRIPAK complex, failed to promote the expression of *CTGF* (Fig. 9b).

Furthermore, we assessed the effects of STRN3 and its two “arms” on the proliferation of HGC-27 cells. In contrast to the inhibitory roles of MST1/2, the overexpression of STRN3 or its two “arms” significantly promoted the proliferation of HGC-27 cells (Fig. 9c and Supplementary Fig. S9). However, the mutants disabled for STRIPAK complex assembly did not increase the cell proliferation or only had a marginal effect (Fig. 9c and Supplementary Fig. S9). Taken together, these results further supported the “two-arm” model of STRIPAK assembly and also confirmed the regulatory role of STRIPAK complex as a whole in Hippo signaling.

### Dynamic assembly of the STRIPAK complex

**Fig. 10:**
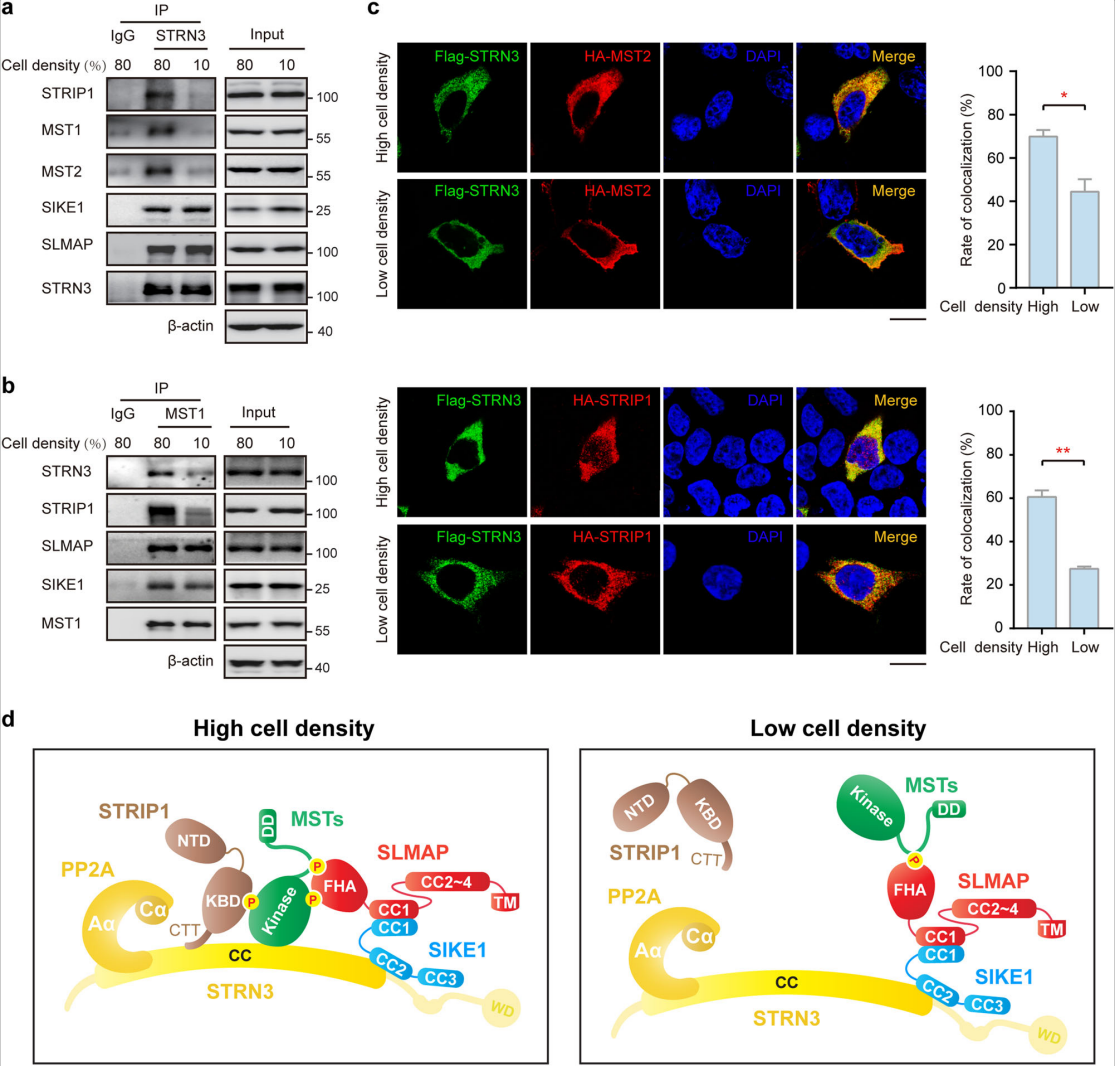
Dynamic assembly of STRIPAK complexes. **a** Co-immunoprecipitation and immunoblot analysis of the endogenous interactions between core STRIPAK components and STRN3 in HEK293FT cells at two different cell densities. **b** Co-immunoprecipitation and immunoblot analysis of the endogenous interactions between core STRIPAK components and MST1 in HEK293FT cells at two different cell densities. **c** Confocal microscope analysis of the colocalization of MST2 or STRIP1 with STRN3 in HEK293FT cells at two different cell densities. The rate of colocalization was quantified and shown (right). **d** Model of the dynamic assembly of the STRIPAK complex induced by different cell densities

Since Hippo signaling is regulated by cell–cell contact^40,41^, we reasoned that cell density might influence the assembly of STRIPAK complexes and thus modulate the activity of Hippo kinase. To test this hypothesis, we first performed endogenous co-IP experiments. Consistent with the previous studies, the phosphorylation levels of MST1 (T183)/MST2 (T180), MOB1 (T35), and YAP1 (S127) were decreased at a lower (10%) cell density when compared with that at a higher (80%) cell confluence (Supplementary Fig. S10a). Moreover, our results showed that the associations of STRN3 with STRIP1 and MST1/2/4 were significantly less at a low cell density than at a high cell density, while its associations with SIKE1 and SLMAP were not affected when the cell density was decreased (Fig. 10a and Supplementary Fig. S10b). Moreover, the interactions of MST1 with STRN3 and STRIP1 were reduced along with a decreased cell confluence, while its interaction with SLMAP and SIKE1 remained unaffected (Fig. 10b). To further verify this dynamic assembly of STRIPAK complexes, we performed confocal microscopic analyses. STRN3 was found strongly colocalized with MST2 and STRIP1 at a high cell confluence (80%), while their colocalization was significantly reduced at a low cell confluence (10%) (Fig. 10c and Supplementary Fig. S10c). Taken together, these results supported a model of the STRIPAK complex with dynamic assembly (Fig. 10d).

## Discussion

The STRIPAK complexes are emerging as important regulators of physiological and pathological processes^3,11^. However, the topological organization of these complexes is still not fully understood. In this study, we dissected in detail the interactions between the major known components of the STRIPAK complexes. We found that PP2Aa/c-bound STRN3 acts as a central scaffold to directly recruit STRIP1, MST kinases, and SIKE1, while STRIP1 directly binds MST kinases and SLMAP binds both SIKE1 and MST kinases. A previous analysis of the results of affinity chromatography coupled with mass spectrometry^2^ led to the proposal of a model in which STRIP1/2 links SLMAP/TRAF3IP3 to STRNs, and SLMAP/TRAF3IP3 further recruits SIKE1/FGFR1OP2. In the current model, STRIP1 does not directly contact either SIKE1 or SLMAP—and STRN3, in addition to directly contacting MST kinases, further utilizes the two “arms”, namely STRIP1 and SIKE1-SLMAP, to recruit MST kinases in a phosphorylation-dependent manner.

The interactions between STRIPAK components are mediated by several kinds of domains. The coiled-coil domain is a ubiquitous protein-interaction domain mediating homo- and heterodimerization of proteins^35,39^. It mediates the assembly of many supramolecular complexes such as the KMN network essential for the attachment of microtubule plus ends to kinetochores^42^ and the SNARE complexes required for intracellular membrane fusion^43^. The coiled-coil domain is also widely present in STRIPAK components such as STRNs, SIKE1/FGFR1OP2, and SLMAP/TRAF3IP3. The coiled-coil region of STRN3 was shown in the current work to not only recruit PP2Aa, STRIP1, and MST kinases, but also to interact directly with the coiled-coil domain of SIKE1. Moreover, SIKE1 and SLMAP also interact with each other through coiled-coil domains. Another two STRIPAK components, CTTNBP2 and its paralog CTTNBP2NL, also contain long coiled coils, which might play roles in recruiting other proteins to the STRIPAK complex.

The FHA domain is another universal protein-binding domain, and it can recognize phosphorylated partners^44,45^. In the STRIPAK complex, SLMAP contains an FHA domain that interacts with phosphorylated kinases. Structural and biochemical analysis demonstrated the conserved phosphopeptide-binding residues of SLAMP to be essential for recruiting kinase components during the assembly of STRIPAK complexes. Recently, two groups reported that the FHA domain of SLMAP directly recognizes multiple autophosphorylated sites of the MST2/Hippo linker region between its kinase and SARAH domains to negatively regulate Hippo signaling^31,32^. In addition to this type of interaction, we found that SLMAP also binds to the kinase domain of MST2, and only to the kinase domain in the case of MST4, suggesting this domain to be a primary site for SLMAP recognition. Interestingly, we identified a novel phosphoprotein-binding domain in STRIP1, namely the KBD. Like SLMAP, the KBD of STRIP1 also directly binds phosphorylated MST kinases. At this stage, it remains unclear which partners the FHA domain of SLMAP and the KBD of STRIP1/2 prefer. Further studies are expected to determine the optimal recognition motifs of these two domains, which should help to identify the new partners of SLMAP and STRIP1/2.

The Hippo signaling pathway plays vital roles in organ-size control, tissue homeostasis, tumorigenesis, immune response, and mechanosensing^28,46–48^. The components of STRIPAK complex, especially SLMAP, have been implicated in Hippo signaling^3,4,6,7,32^. In the present study, we found that an intact STRIPAK complex is required for the regulation of Hippo signaling. Mutants of STRIPAK components disrupting STRIPAK assembly failed to induce YAP activity and cancer cell proliferation. In particular, we discovered SIKE1 as a key adaptor that recruits MST-bound SLMAP to PP2A-bound STRN, forming a molecular arm MST-SLMAP-SIKE1-STRN-PP2Aa/c. SIKE1 functions to bridge SLMAP through CC1 on one hand and STRN3 through CC2 on the other. SIKE1 CC2 alone exists as a homotetramer (dimer of dimer), but forms a 2:1 heterotrimer with STRN3. Meanwhile, SIKE1 CC1 can form a 2:2 hetrotetramer with SLMAP CC1. Thus, one STRN3 molecule may recruit two SIKE1 molecules, which in turn can further recruit two SLMAP molecules bound with two MST molecules. Considering the dimerization of STRN3 itself and the dimerization of recruited MST kinases, the assembly of STRIPAK complex would be highly complicated and dynamic, possibly with network- and context-dependent features. The protein of full-length SIKE1 is heterogeneous (data not shown), and the binding of STRN3 or SLMAP may stabilize SIKE1 with a certain conformation.

Interestingly, our data argue for a dynamic assembly model of the STRIPAK complex in response to cell density (Fig. 10d), which may contribute to the regulation of GCK kinase activity, especially MST1/2 in Hippo signaling. According to this model, when the cell density is high, MST kinases are phosphorylated (active), and STRIP1 and SLMAP/SIKE1 are able to recognize these phosphorylated kinases and thus help to stabilize their association with STRN3. In contrast, under conditions of low cell density, STRIP1 tends to disassociate from the complex, while dephosphorylated (inactive) MST kinases turns away from the STRN-bound PP2A core enzyme. It is very likely that other cellular inputs such as mechanical, nutritional, and metabolic stress may also serve as regulatory cues for the dynamic assembly of the STRIPAK complex. In this regard, STRIPAK may act as a biosensor upstream of the Hippo pathway to integrate various signals.

STRIP2 is a paralog of STRIP1, and a previous study identified two isoforms for STRIP2, with isoform 2 lacking the very C terminus required for its association with PP2A^33^. STRIP1 and isoform 1 of STRIP2 were each shown in a co-immunoprecipitation assay of that study to associate with PP2Ac, but isoform 2 of STRIP2 did not. Isoform 2 of STRIP2 has been proposed to antagonize the function of STRIP1 to promote breast cancer metastasis. Our current study indicated that STRIP1 associates with PP2Ac through its direct interaction with STRNs. Moreover, we showed the CTT of STRIP1 (which is highly conserved in sequence to the CTT of isoform 1 of STRIP2) to be the binding site for STRN3. Thus, isoform 2 of STRIP2, lacking the CTT, cannot bind to STRN3 and hence neither to PP2Ac. Meanwhile, this CTT of STRIP is not essential for binding MST kinases. Thus, isoform 2 of STRIP2 is still able to bind MST kinases, and would hence compete with STRIP1 for kinase recruitment and therefore result in the antagonizing effects mentioned above. The coiled-coil domain of STRN3 is the binding region for STRIP1. Our preliminary data suggested the N-terminal region of the STRN3 coiled coil close to the caveolin-binding site^49^ to be required for its interaction with STRIP1. Therefore, the recruitment of STRIP1 by STRNs might regulate the binding of caveolin to STRNs, and the interaction between caveolin and STRNs might also affect the binding of STRIP1 to STRNs. On the other hand, the C-terminal region of the STRN3 coiled coil, which is also thought to be responsible for recruiting calmodulin^50,51^, appears necessary for the ability of STRN3 to bind SIKE1. Thus, it is also likely that this binding of SIKE1 to STRNs interferes with the function of the STRN-calmodulin complex, and calmodulin might also regulate the recruitment of SIKE1 by STRNs. Further investigation is needed to verify the context-dependent assembly of STRIPAK complexes.

In addition to STRN3, STRIP1, and SLMAP, CCM3 has also been reported to directly interact with GCKIII kinases and thus recruit them to STRNs. We recently found MOB4 to be another phosphorylation-dependent binding partner for GCKs^52^. Therefore, the STRIPAK complexes could utilize multiple proteins to recruit and regulate the kinase components. Given the important roles of GCKs in development, homeostasis, immune response, tumorigenesis, and cancer metastasis^14,15,28,53^, STRIPAK complexes would be expected to be involved in these biological processes through their regulating the activity of GCKs.

In addition to their immediate relevance to the Hippo signaling pathway, STRIPAK complexes or their components have also been reported to regulate the phosphorylation of other kinases or non-kinase proteins. SIKE1 associates with TBK1 and IKKε to regulate their kinase activities and antiviral responses^26^. The phosphatase part of the *Drosophila* STRIPAK complex has been shown to regulate the phosphorylation of CLOCK in *Drosophila*^54^. Thus, STRIPAK complexes can modulate the phosphorylation and activity of diverse proteins with the aid of various adaptors, with only some of these adaptors having been identified. In the current work, we provided a topological model of STRIPAK complexes with high-resolution substructures and dynamic features, which may benefit further structural and functional studies relevant to Hippo signaling, development, cancer, immunity, as well as mechanobiology.

## Materials and methods

### Plasmids and antibodies

Constructs of full-length or truncated human STRN3, PP2Aa, SIKE1, FGFR1OP2, SLMAP, TRAF3IP3, MST2, MST4, and OSR1 were cloned into modified pET28a vectors with an N-terminal 6 × His, 6 × His-SUMO, GST, or MBP tag followed by a tobacco etch virus (TEV)^55^ protease-cleavage site or the pGEX4T/6P-1 vector with an N-terminal GST tag followed by a thrombin or PreScission protease-cleavage site. Full-length PP2Ac and STRIP1 were cloned into the pFastBac vectors and expressed in High Five insect cells as TEV-cleavable His-tagged proteins. For PP2Ac, an uncleavable HA tag was attached to its N terminus as previously described^56^.

For cellular assays, Flag-tagged STRN3, MST2, and SLMAP were cloned into a pcDNA3.1 vector, and HA-tagged STRIP1 and SIKE1 were cloned into a pcDNA3.0 vector. All mutants were generated by carrying out site-directed mutagenesis. All constructs were verified by performing DNA sequencing.

Antibody specific for human MST4 was produced by Shanghai Immune Biotech Co., Ltd., and has been described previously^57^. An antibody specific for Flag (F3165) was obtained from Sigma; antibodies to HA (rabbit, #3724), MST1 (rabbit, #3682), MOB1 (#13730), p-MOB1 (T35, #8699), p-YAP (S127, #13008), MST1 (#3682), and p-MST1 (T183)/p-MST2 (T180) (#3681) were from Cell Signaling Technology; antibodies to YAP (sc-101199), SLMAP (sc-100957), HA (mouse, sc-7392), MST1 (mouse, sc-515051), and MST2 (sc-130405) were from Santa Cruz; an antibody to STRN3 (#PA5-31368) was from Invitrogen; and antibodies to SIKE1 (ab121860) and STRIP1 (ab199851) were from Abcam.

### siRNA

Duplexes for the siRNA targeting of MST2, SIKE1, SLMAP, STRN3, STRIP1, and control siRNA (n.c.) were synthesized by Genepharma (Shanghai, China). The siRNA used in this work was a mixture of two independent ones. The siRNA sequences are as follows:

siMST2-1: 5′-GCUGGUCAGUUAACAGAUATT-3′ (F), 5′-UAUCUGUUAACUGACCAGCTT-3′ (R);

siMST2-2: 5′-CAGAAGCUAUGGAGAUCAATT-3′ (F),5′-UUGAUCUCCAUAGCUUCUGTT-3′ (R);

siSIKE1-1: 5′-CUUCGAGAAUUAUUGUCCATT-3′ (F), 5′-UGGACAAUAAUUCUCGAAGTT-3′ (R);

siSIKE1-2: 5′-GCAGAGGUAUCAAGAGGAUTT-3′ (F), 5′-AUCCUCUUGAUACCUCUGCTT-3′ (R);

siSLMAP-1: 5′-GGAUGAGCAAGACCUAAAUTT-3′ (F), 5′-AUUUAGGUCUUGCUCAUCCTT-3′ (R);

siSLMAP-2: 5′-CCCAAUUGCAGAGGUUACATT-3′ (F), 5′-UGUAACCUCUGCAAUUGGGTT-3′ (R);

siSTRN3-1: 5′-GCCAGUUAACGUGGAAGCATT-3′ (F), 5′-UGCUUCCACGUUAACUGGCTT-3′ (R);

siSTRN3-2: 5′-GGGGAUACUGCACUACAUCTT-3′ (F), 5′-GAUGUAGUGCAGUAUCCCCTT-3′ (R);

siSTRIP1-1: 5′-CAGCATCAAAGTGATTCGCAA-3′ (F), 5′-UUGCGAAUCACUUUGAUGCUG-3′ (R); and

siSTRIP1-2: 5′-CACGTCGATTGCAGAGGTCCA-3′ (F), 5′-UGGACCUCUGCAAUCGACGUG-3′ (R).

### Cells

HEK293FT and HGC-27 cells were obtained from Shanghai Life Academy of Sciences cell library (Shanghai, China). HEK293FT cells were maintained in Dulbecco's modified Eagle's medium and HGC-27 cells were cultured in RPMI 1640 medium. All cells were maintained in the culture medium supplemented with 10% heat-inactivated fetal bovine serum, 100 U/mL penicillin, and 100 μg/mL streptomycin at 37 °C with 5% CO_2_ in a humidified incubator (ThermoFisher Scientific, Waltham, MA, USA).

### Protein expression and purification

All prokaryotic constructs were expressed in *Escherichia coli* (*E. coli*) BL21 (DE3) CodonPlus cells. Protein expression was induced by using 0.25 mM isopropyl β-D-thiogalactopyranoside at OD_600_ = 1.0 in Terrific Broth medium, and cells were cultured for an additional 16 h at 18 °C. Cells were harvested by subjecting to centrifugation and stored at −20 °C. The selenomethionine (Se-Met)-labeled SIKE1 CC2 was expressed in *E.coli* BL21 (DE3) CodonPlus cells cultured in M9 medium containing an amino acid supplement (lysine, phenylalanine, and threonine to a final concentration of 100 mg/L; isoleucine, leucine, and valine to 50 mg/L, and DL-Se-Met (ACROS) to 60 mg/L). For the baculovirus-mediated expression of PP2Ac and STRIP1, proteins were expressed according to the instructions of Bac-to-Bac^®^ Baculovirus Expression System (Invitrogen). Baculovirus was produced using *Spodoptera frugiperda* Sf9 cells and proteins were expressed using High Five cells. All insect cells were cultured in ESF 921 Insect Cell Culture Medium (Expression Systems, 96-001-01) supplemented with 100 U/mL penicillin and 100 μg/mL streptomycin at 27 °C. For baculovirus-mediated expression, High Five cells at a density of 2.5 × 10^6^ cells/mL were infected with fresh baculovirus and cultured at 27 °C for 72 h before harvesting.

All purification procedures were performed at 4 °C unless otherwise stated. Purification of PP2Ac was performed following the procedure described previously^56^. Harvested *E. coli* cells expressing 6 × His-tagged or 6 × His-SUMO-tagged proteins were resuspended in lysis buffer A (20 mM HEPES (pH 7.5), 500 mM NaCl, 5% glycerol, 5 mM β-mercaptoethanol, and 20 mM imidazole), whereas those expressing GST- or MBP-tagged proteins were resuspended in lysis buffer B (20 mM HEPES (pH 7.5), 500 mM NaCl, 5% glycerol, and 5 mM β-mercaptoethanol). Harvested High Five cells expressing His-STRIP1 were resuspended in lysis buffer B supplemented with the protease inhibitor cocktail (EDTA-free, Roche) and 1 mM phenylmethylsulfonyl fluoride. Both the *E. coli* and High Five cells were lysed by a high-pressure homogenizer (UH-06; Union-Biotech, Shanghai, China) at 800 bar. Cell debris was removed by subjecting the lysate to centrifugation at 18,000 rpm for 40 min. The soluble fractions for His- and His-SUMO-tagged proteins were applied to Ni Sepharose (GE Healthcare) pre-equilibrated with lysis buffer A, and the soluble fractions for GST- or MBP-tagged proteins were applied to glutathione Sepharose (GE Healthcare) or amylose resin (New England Biolabs) columns pre-equilibrated with lysis buffer B, respectively. After washing with the corresponding lysis buffer, target proteins were eluted with buffer B supplemented with 300 mM imidazole for the His-/His-SUMO-tagged proteins, 20 mM reduced glutathione for the GST-tagged proteins, or 20 mM maltose for the MBP-tagged proteins. To obtain untagged proteins, the different tags were removed by treating the tagged proteins with a homemade His-tagged TEV protease or thrombin (GE Healthcare) protease. Protease-treated His-/His-SUMO-tagged proteins were re-applied to Ni Sepharose to remove the cleaved His/His-SUMO tag and TEV protease. PP2Aa was further purified by ion-exchange chromatography (Mono Q, GE Healthcare) followed by gel filtration chromatography (HiLoad Superdex 75/200, GE Healthcare) with 20 mM HEPES (pH 7.5), 100 mM NaCl, and 1 mM dithiothreitol as the running buffer. The rest of the proteins were further purified by gel filtration chromatography (HiLoad 16/60 Superdex 75/200 pg, GE Healthcare) without ion-exchange chromatography. The Se-Met-labeled SIKE1 CC2 was purified following the same procedure as with the native protein. The purified proteins were then concentrated and stored at −80 °C.

### Crystallization, structural determination, and refinement

For crystallization, the purified native and Se-Met-labeled SIKE1 CC2 proteins were concentrated to 12 mg/mL and 15.8 mg/mL, respectively. For SIKE1 CC2-STRN3 complex, SIKE1 CC2 (12 mg/mL) was mixed with STRN3 peptide (residues 166–190) at 1:1.2 molar ratio before crystallization. The reconstituted SIKE1 CC1-SLMAP CC1 complex was purified by mixing each purified protein in 1:1 molar ratio followed by gel filtration chromatography (HiLoad 16/60 Superdex 75 pg); proteins of SIKE1 CC1-SLMAP CC1 complex were concentrated to 10 mg/mL before crystallization. Crystallization trials were carried out at 16 °C using the sitting-drop vapor diffusion method. Crystals of the native apo SIKE1 CC2 were grown in a reservoir solution consisting of 0.1 M Tris (pH 8.5) and 0.7 M ammonium tartrate dibasic. Crystals of the Se-Met-labeled apo SIKE1 CC2 were grown in a reservoir solution consisting of 1.0 M (NH4)_2_HPO_4_ and 0.1 M acetate (pH 4.5). Crystals of the SIKE1 CC2-STRN3 complex were grown in a reservoir solution consisting of 35% (v/v) 2-Methyl-2, 4-pentanediol (MPD) and 0.1 M imidazole (pH 7.5). Crystals of the SIKE1 CC1-SLMAP CC1 complex were grown in a reservoir solution consisting of 0.1 M sodium citrate (pH 5.5) and 15% (w/v) PEG 6000. The crystals were cryoprotected with the corresponding protectants and flashcooled in liquid nitrogen before data collection. Diffraction data of native SIKE1 CC2 were collected at beamline BL17U, Shanghai Synchrotron Radiation Facility (SSRF) of China, and processed using HKL2000^58^. Diffraction data of Se-Met-labeled SIKE1 CC2, SIKE1 CC2-STRN3 complex, and SIKE1 CC1-SLMAP CC1 complex were collected at beamline BL18U1, SSRF of China, and processed using HKL3000. The structure of apo SIKE1 CC2 was solved by the single-wavelength anomalous diffraction^59^ method using the anomalous signal from Se. Structures of SIKE1 CC2-STRN3 and SIKE1 CC1-SLMAP CC1 complexes were solved by molecular replacement^60^ using SIKE1 CC2 as the model. Automated model building of SIKE1 CC2 was performed with CCP4 i2^61,62^, molecular replacement was performed using phenix, and the structure was refined using phenix.refine and COOT^63–65^.

### GST and MBP pulldown assays

GST-tagged prey proteins, bait proteins, and glutathione Sepharose were mixed at 4 °C for 1 h in 20 mM HEPES (pH 7.5) and 100 mM NaCl. MBP-tagged prey proteins, bait proteins, and amylose resin were mixed at 4 °C for 1 h in 20 mM HEPES (pH 7.5) and 100 mM NaCl. The beads were washed two times with the same buffer. Proteins were eluted with 20 mM HEPES (pH 7.5), 100 mM NaCl, and 20 mM reduced glutathione or maltose. The input and output samples were loaded on a sodium dodecyl sulfate-polyacrylamide gel electrophoresis (SDS-PAGE) gel and detected by applying Coomassie Brilliant Blue staining or western blot.

### Isothermal titration calorimetry assay

An ITC assay was performed in an iTC200 microcalorimeter (Microcal, Piscataway, NJ, USA) at 25 °C. The proteins were in the buffer containing 20 mM HEPES (pH 7.5) and 100 mM NaCl. Data were analyzed using Origin software (OriginLab, Northampton, MA, USA) provided with the instrument.

### Bio-layer interferometry experiment

A bio-layer interferometry experiment was performed using Octet Red 96 (ForteBio) as previously described^66^. Biotinylated proteins were immobilized on streptavidin biosensors and then incubated with the corresponding binding partners in kinetics buffer (20 mM HEPES (pH 7.5), 100 mM NaCl, 0.01% bovine serum albumin, and 0.001% Triton X-100). All binding experiments were performed at 25 °C. Data were analyzed using Octet Data Analysis Software 7.0 (ForteBio).

### Immunoprecipitation and immunoblot analysis

For immunoprecipitation experiments, whole-cell extracts of HEK293FT cells were collected 24 h after the cells were transfected. The extracts were specifically obtained by lysing the cells with RIPA buffer (150 mM NaCl, 100 mM Tris (pH 8.0), 1% Triton X-100, 5 mM EDTA, and 10 mM NaF) supplemented with 1 mM phenylmethylsulfonyl fluoride and the protease inhibitor cocktail (Sigma). The lysate was subjected to centrifugation at 12,000 rpm for 20 min at 4 °C, and the resulting supernatants were collected and incubated overnight with indicated antibodies together with Protein A/G beads (Santa Cruz). After incubation, the beads were washed and then eluted with SDS loading buffer and boiled. For immunoblot analysis, immunoprecipitates or whole-cell extracts were run on an SDS-PAGE gel, and the separated bands from the gel were transferred onto polyvinylidene difluoride membranes and then identified with the indicated antibodies.

### Real-time PCR

Real-time PCR was performed on a Two-Step Real-Time PCR System using the comparative Ct quantization method. qPCR SYBR^®^ Green Master Mix (Yeasen, Shanghai, China) was used to detect and quantify the expression level of the target gene. GAPDH was used as an internal control. The primers used were as follows:

CTGF:

5′-AAAAGTGCATCCGTACTCCCA-3′ (F),

5′-CCGTCGGTACATACTCCACAG-3′ (R);

GAPDH:

5′-GGCATCCTGGGCTACACTGA-3′ (F),

5′-GAGTGGGTGTCGCTGTTGAA-3′ (R).

F, forward; R, reverse.

### Cell proliferation assay

Cell proliferation assay was performed according to the protocol of the CellTiter-Lumi™ Plus Luminescent Cell Viability Assay Kit (Beyotime, Shanghai, China). Briefly, an equal volume of CellTiter-Lumi™ Plus Reagent added to the cell culture medium was added to the wells and mixed for 2 min at room temperature on an orbital shaker to induce cell lysis. After an additional 10 min incubation at room temperature, the luminescent signals were measured.

### Confocal imaging and structured illumination microscopy

Cells were grown on glass bottom cell culture dishes (NEST, 801001), washed once with phosphate-buffered saline, and fixed in 4% paraformaldehyde. After permeabilization, the cells were blocked with 5% bovine serum albumin and then incubated with primary antibodies. After three separate washes, the cells were incubated with Alexa Fluor-conjugated secondary antibodies and then stained with 4′, 6-diamidino-2-phenylindole. Images were captured using a Leica laser scanning confocal microscope (Leica SP5) or a structured illumination microscope (Leica OMX).

### Statistical analysis

Statistical analysis was performed with the SAS statistical software package (9.1.3) and GraphPad Prism 7.0. Data are presented as means ± SD. Student’s *t* test was used for continuous variables. *p* Values less than 0.05 were considered statistically significant.

### Accession codes

The structural coordinates of SIKE1 CC2, SIKE1 CC2-STRN3 complex, and SIKE1 CC1-SLMAP CC1 complex were deposited in the PDB under the codes 6AKK, 6AKL, and 6AKM, respectively.

## Supplementary information

Supplementary Information

## Data Availability

The data that support the findings of this study are available from the corresponding authors upon reasonable request.

## Electronic supplementary material

**Supplementary Information** accompanies the paper at (10.1038/s41421-018-0077-3).
