# Supplementary material for: Architecture, substructures, and dynamic assembly of STRIPAK complexes in Hippo signaling
Source: Cell Discov. 2019 Jan 8;5:3. doi: 10.1038/s41421-018-0077-3 (PMC6323126; doi:10.1038/s41421-018-0077-3)
Supplement: Supplementary file 1 — Supplementary Information [file 41421_2018_77_MOESM1_ESM.pdf]

Supplementary Information

a

Classification	Members
Phosphotase components	PP2Aa<br>PP2Ac<br>STRNs (STRN, STRN3, STRN4)
Kinase components	GCKII (MST1/2)<br>GCKIII (MST3/4, STK25)<br>GCKIV (MINK1, TNIK, MAP4K4)
Other components	CCM3<br>STRIP1/2<br>MOB4<br>SLMAP/TRAF3IP3<br>SIKE1/FGFR1OP2<br>CTTNBP2/CTTNBP2NL

b

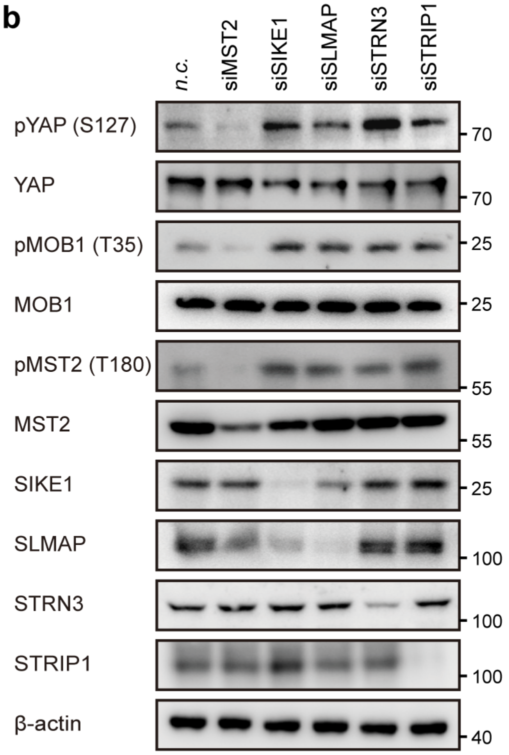

Supplementary Figure S1. Major components of the STRIPAK complex. (**a**) Summarization and classification of the major components in STRIPAK complex. (**b**) The intact figure corresponding to Figure 1b.

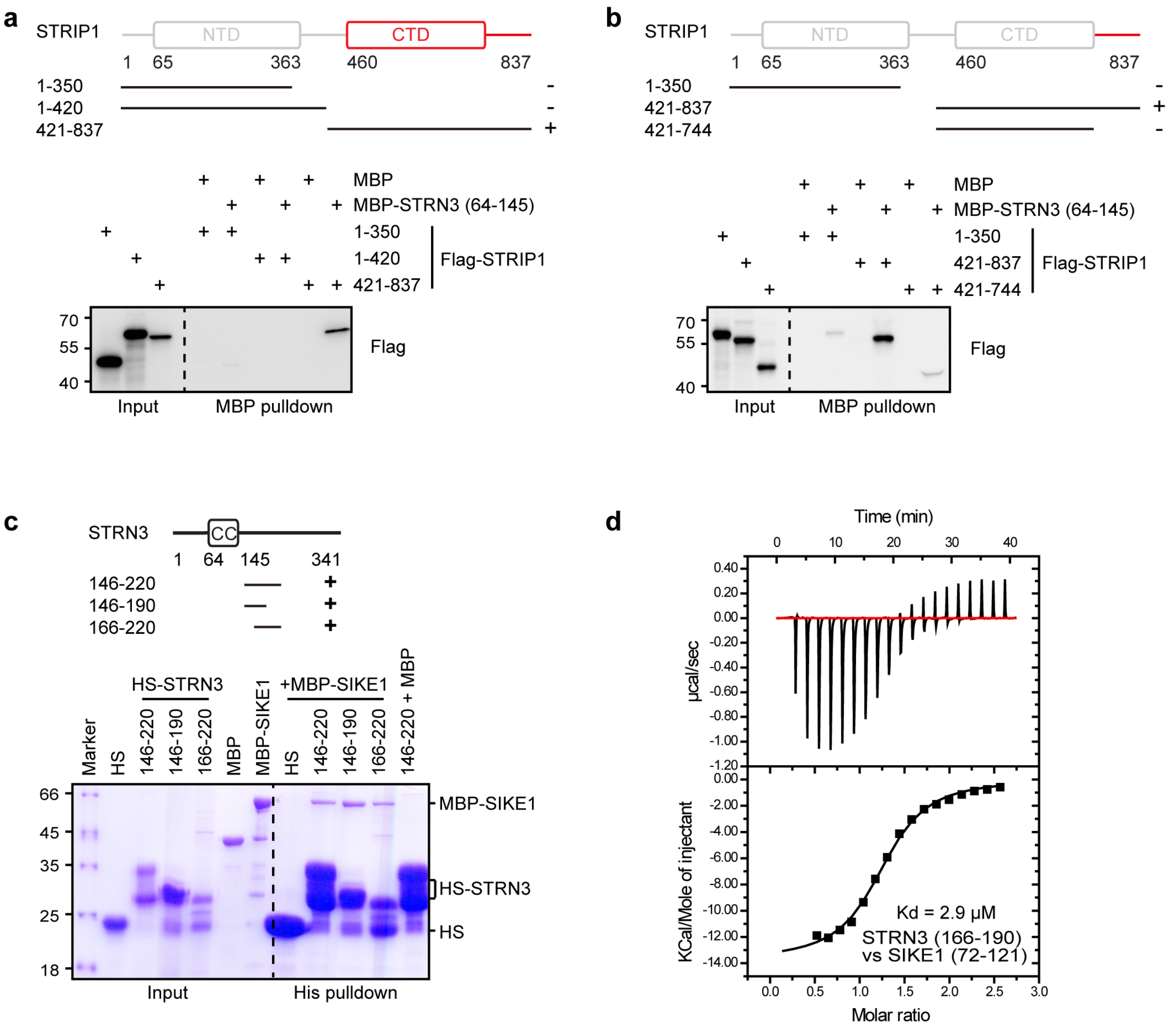

Supplementary Figure S2. Domain mapping of STRN3 and STRIP1/SIKE1. (**a**) and (**b**) MBP-pulldown-based determination of the STRN3-binding domain of STRIP1. Fragments of Flag-STRIP1 were obtained using an *in vitro* cell-free system. The input and output samples were loaded on an SDS-PAGE gel followed by immunoblot analysis. (**c**) Mapping of SIKE1 binding region on STRN3. HS, His-SUMO tag. (**d**) ITC analysis of the interaction between SIKE1 (72-121) and a STRN3 peptide (residues 166-190).

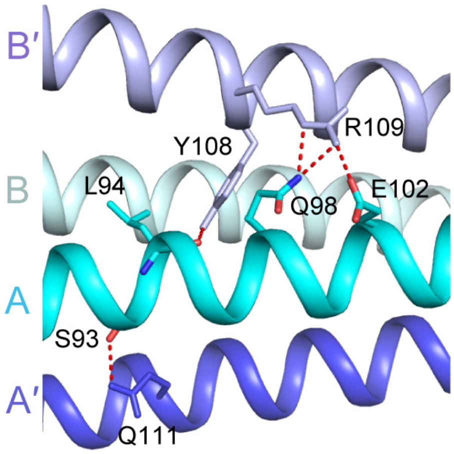

Supplementary Figure S3. Interaction analysis of apo SIKE1 CC2 interface. Polar Interactions at the tetrameric interface of SIKE1 CC2. ***Notes***: The red dash lines represent hydrogen bonds or salt bridge. The interface residues are labeled and highlighted by stick model.

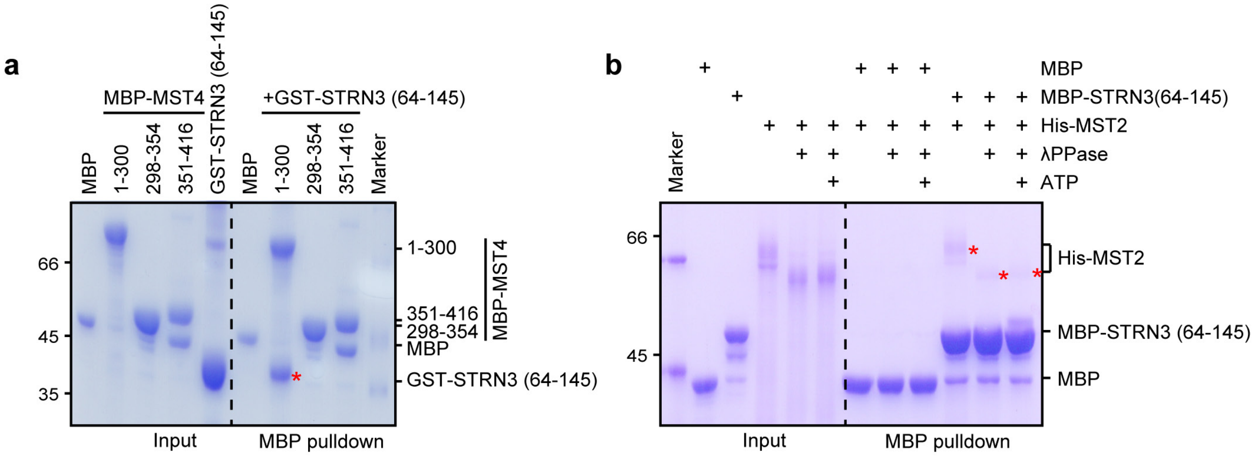

Supplementary Figure S4. Analysis of the interaction between STRN3 and MST2/4. (**a**) MBP-pulldown-based determination of the STRN3-binding domain of MST4. The input and output samples were loaded on an SDS-PAGE gel followed by staining this gel with CBB. (**b**) Pulldown analysis of the interaction between MST2/pMST2 and STRN3. Purified protein of MST2 was first dephosphorylated by lambda-phosphatase (λPPase) or further autophosphorylated in the presence of ATP before use. The input and output samples were loaded on an SDS-PAGE gel followed by staining this gel with CBB.

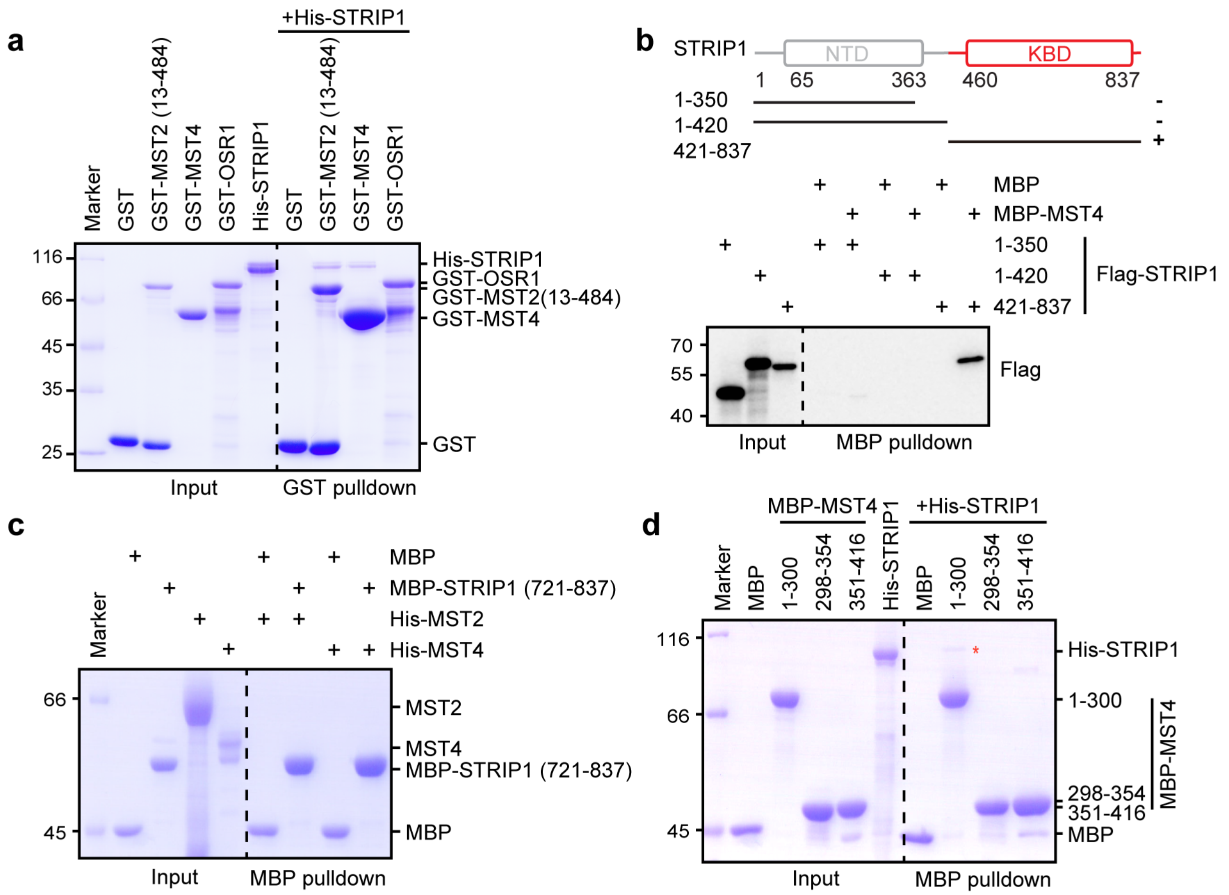

Supplementary Figure S5. Analysis of the interaction between STRIP1 and MST2/4. (**a**) GST-pulldown analysis of interactions between STRIP1 and MST2, MST4 or OSR1. The input and output samples were loaded on an SDS-PAGE gel followed by staining this gel with CBB. (**b**) MBP-pulldown-based determination of the MST4-binding domain of STRIP1. Fragments of Flag-STRIP1 were obtained using an *in vitro* cell-free system. The input and output samples were loaded on an SDS-PAGE gel followed by immunoblot analysis. (**c**) MBP-pulldown analysis of the interaction between the STRIP1 C-terminal tail and MST2/4. The input and output samples were loaded on an SDS-PAGE gel followed by staining this gel with CBB. (**d**) MBP-pulldown-based determination of the STRIP1-binding domain of MST4. The input and output samples were loaded on an SDS-PAGE gel followed by staining this gel with CBB.

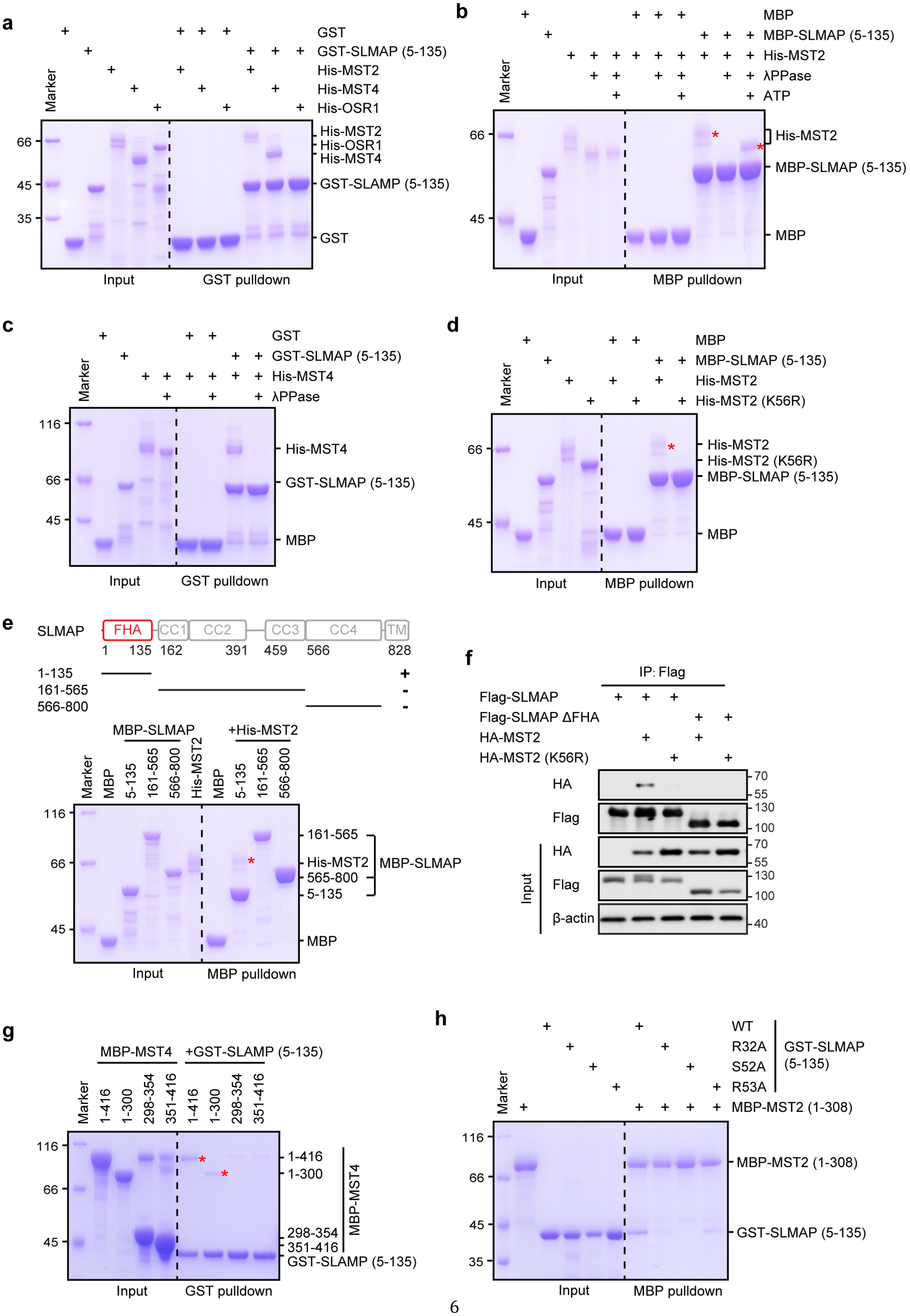
a
GST
GST-SLMAP (5-135)
His-MST2
His-MST4
His-OSR1
Marker
66
45
35
His-MST2
His-OSR1
His-MST4
GST-SLAMP (5-135)
GST
Input
GST pulldown
b
MBP
MBP-SLMAP (5-135)
His-MST2
λPPase
ATP
Marker
66
45
His-MST2
MBP-SLMAP (5-135)
MBP
Input
MBP pulldown
c
GST
GST-SLMAP (5-135)
His-MST4
λPPase
Marker
116
66
45
His-MST4
GST-SLMAP (5-135)
MBP
Input
GST pulldown
d
MBP
MBP-SLMAP (5-135)
His-MST2
His-MST2 (K56R)
Marker
66
45
His-MST2
His-MST2 (K56R)
MBP-SLMAP (5-135)
MBP
Input
MBP pulldown
e
SLMAP
FHA
CC1
CC2
CC3
CC4
TM
1 135 162 391 459 566 828
1-135
161-565
566-800
+
-
-
MBP-SLMAP
+His-MST2
Marker
MBP
5-135
161-565
566-800
His-MST2
116
66
45
161-565
His-MST2
565-800
5-135
MBP-SLMAP
MBP
Input
MBP pulldown
f
IP: Flag
Flag-SLMAP
Flag-SLMAP ΔFHA
HA-MST2
HA-MST2 (K56R)
HA
Flag
HA
Flag
β-actin
Input
70
55
130
100
40
g
MBP-MST4
+GST-SLAMP (5-135)
Marker
1-416
1-300
298-354
351-416
116
66
45
1-416
1-300
298-354
351-416
GST-SLAMP (5-135)
MBP-MST4
Input
GST pulldown
h
WT
R32A
S52A
R53A
GST-SLMAP (5-135)
MBP-MST2 (1-308)
Marker
116
66
45
35
MBP-MST2 (1-308)
GST-SLMAP (5-135)
Input
MBP pulldown

Supplementary Figure S6. Analysis of the interaction between SLMAP and MST2/4. (**a**) GST-pulldown analysis of interactions between SLMAP (FHA) and MST2, MST4 or OSR1. The input and output samples were loaded on an SDS-PAGE gel followed by staining this gel with CBB. (**b**) Pulldown analysis of the interaction between MST2/pMST2 and SLMAP. Purified protein of MST2 was first dephosphorylated using lambda-phosphatase (λPPase) or further autophosphorylated in the presence of ATP before use. The input and output samples were loaded on an SDS-PAGE gel followed by staining this gel with CBB. (**c**) Pulldown analysis of interactions between MST4/pMST4 and SLMAP (FHA). Purified MST4 protein was first dephosphorylated by lambda-phosphatase (λPPase) before use. The input and output samples were loaded on an SDS-PAGE gel followed by staining this gel with CBB. (**d**) MBP-pulldown analysis of the interaction between SLMAP and the wildtype or kinase-inactive mutant form (K56R) of MST2. The input and output samples were loaded on an SDS-PAGE gel followed by staining this gel with CBB. (**e**) MBP-pulldown-based determination of the MST2-binding domain of SLMAP. The input and output samples were loaded on an SDS-PAGE gel followed by staining this gel with CBB. (**f**) Co-immunoprecipitation and immunoblot analysis of SLMAP and MST2 in HEK293FT cells. ΔFHA: deletion of residues 1-135. (**g**) GST-pulldown-based determination of the SLMAP-binding domain of MST4. The input and output samples were loaded on an SDS-PAGE gel followed by staining this gel with CBB. (**h**) MBP-pulldown analysis of the interactions between the MST2 kinase domain and the indicated SLMAP (FHA) mutants. The input and output samples were loaded on an SDS-PAGE gel followed by staining this gel with CBB.

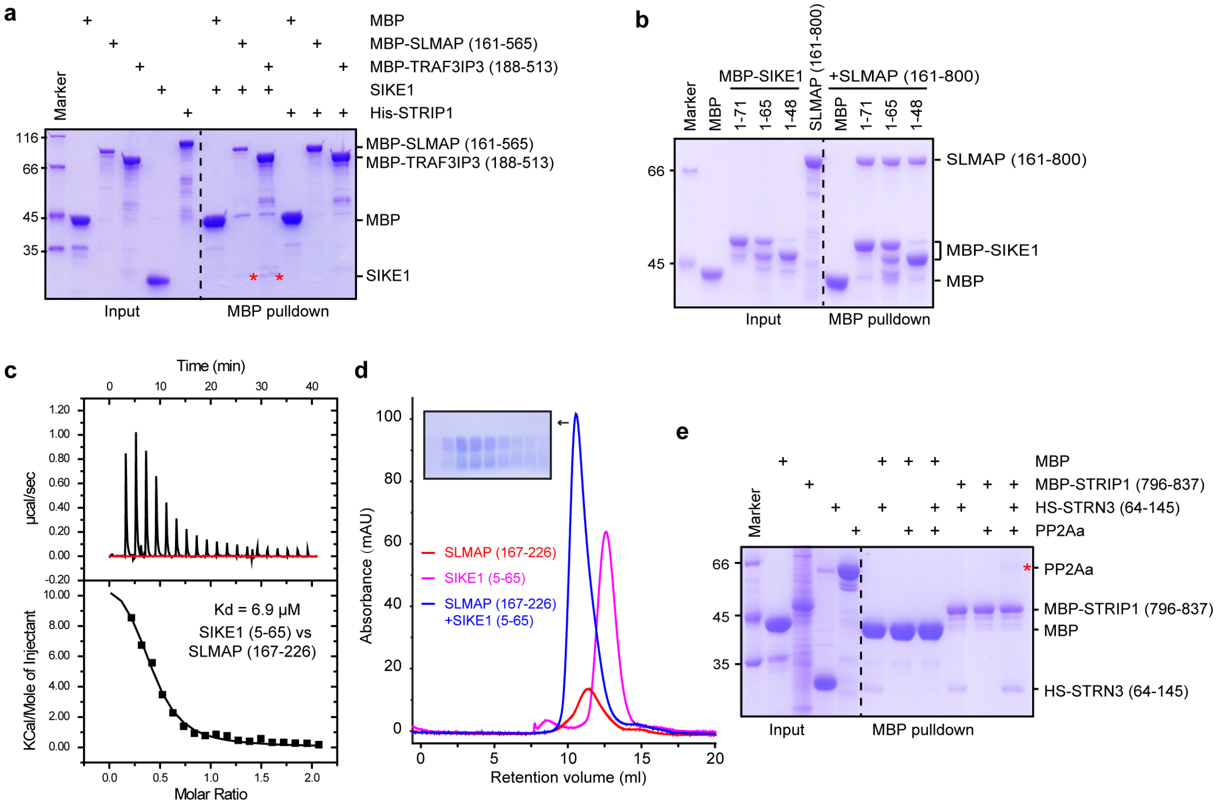

Supplementary Figure S7. Analysis of interactions between SLMAP/TRAF3IP3, SIKE1 and STRIP1/STRN3/PP2Aa. (**a**) MBP-pulldown analysis of the interaction between SLMAP/TRAF3IP3 and SIKE1 or STRIP1. TRAF3IP3: a homolog of SLMAP. The input and output samples were loaded on an SDS-PAGE gel followed by staining this gel with CBB. (**b**) Mapping of SLMAP binding region on SIKE1. (**c**) ITC analysis of the interaction between SIKE1 (5-65) and a SLMAP (167-226). (**d**) UV traces of SLMAP (167-226, red line), SIKE1 (5-65, pink line) or SLMAP (167-226) and SIKE1 (5-65) complex (blue line) fractionated on a Superdex 75 10/300 GL gel filtration column. The corresponding SDS-PAGE gel of the elution fractions of SLMAP (167-226)-SIKE1 (5-65) complex was shown. (**e**) MBP-pulldown analysis of the interaction between STRIP1 and PP2Aa in the absence or presence of STRN3. The input and output samples were loaded on an SDS-PAGE gel followed by staining this gel with CBB.

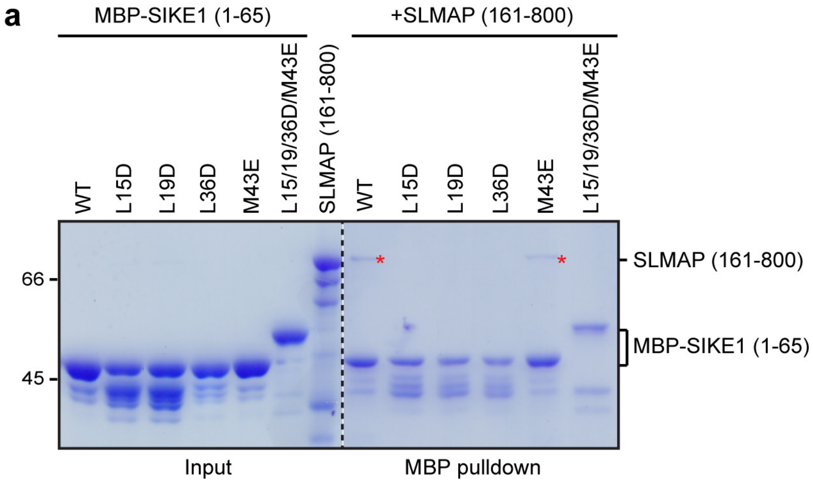

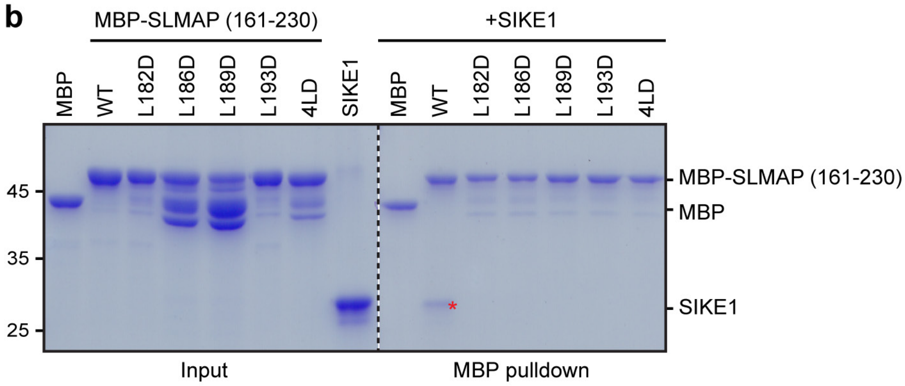

Supplementary Figure S8. Mutational analysis of SIKE1-SLMAP interface. (**a**) and (**b**) Pulldown analyses between wildtype or mutant forms of SIKE1 and SLMAP. The input and output samples were loaded on an SDS-PAGE gel followed by staining this gel with CBB.

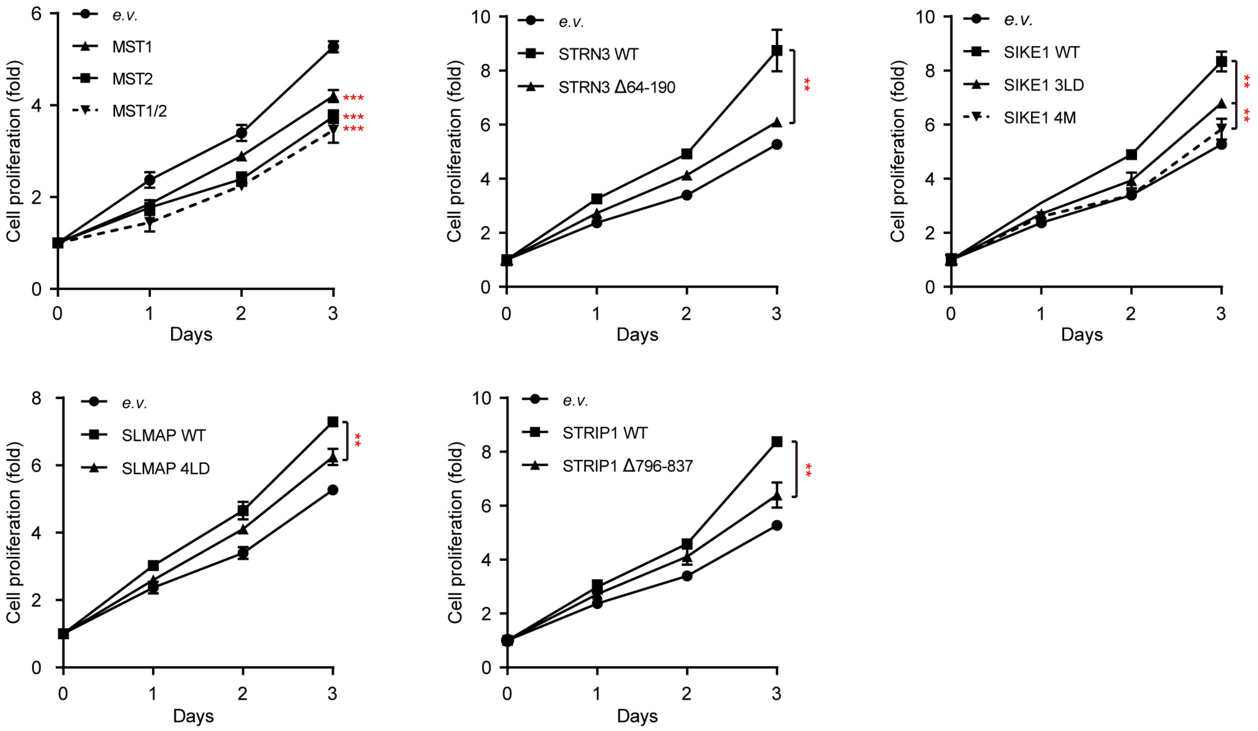

Supplementary Figure S9. Cell proliferation assay results. Cell proliferation of HGC-27 cells transfected with the indicated plasmids was determined by CellTiter assay. Bar graphs represent the means ± SD. Experiments we repeated three times. Unpaired *t* tests were used to compare the difference between two groups. * significant relative to wildtype, $p < 0.05$, ** $p < 0.01$, *** $p < 0.001$.

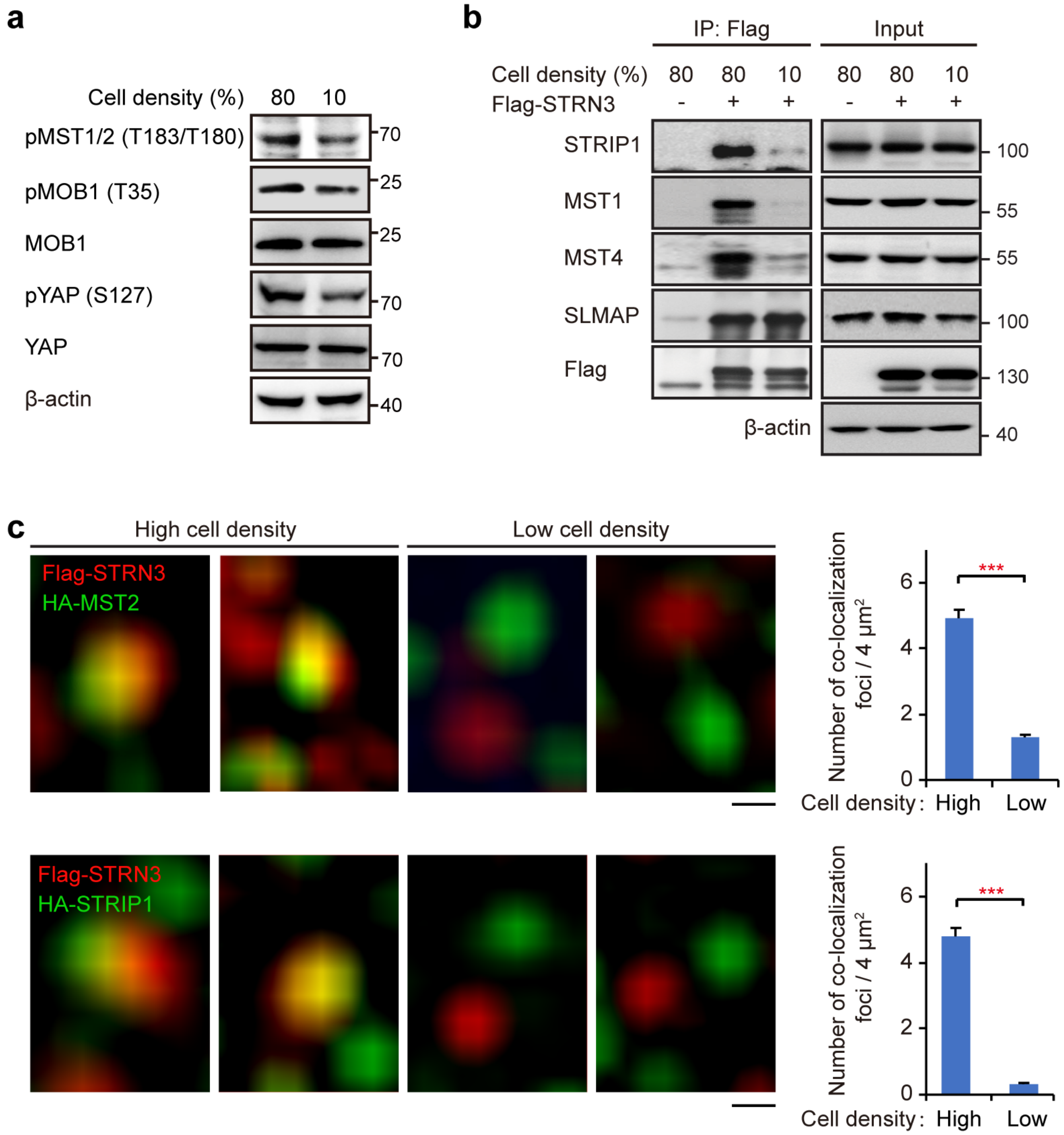

Supplementary Figure S10. Dynamic assembly of the STRIPAK complex. (**a**) Phosphorylation levels of MST1/2, MOB1 and YAP1 in HEK293FT cells at different cell density was detected with the indicated antibodies. (**b**) Co-immunoprecipitation and immunoblot analysis of the interactions between core STRIPAK components and Flag-STRN3 in HEK293FT cells at two different cell densities. (**c**) Structured illumination microscopy (SIM) analysis of the co-localization of MST2 or STRIP1 with STRN3 in HEK293FT cells at two different cell densities. Scale bars, 0.1 μm.

Supplementary Table S1

Data collection and refinement statistics of SIKE1 CC2

	Native	Se-Met
Data collection		
Wavelength (Å)	0.97915	0.97853
Space group	$C\,2\,2\,2_1$	$C\,2\,2\,2_1$
Cell dimensions		
a, b, c (Å)	42.80, 110.87, 46.44	43.00, 110.88, 46.59
α, β, γ (°)	90, 90, 90	90, 90, 90
Resolution (Å)	50-1.50 (1.53-1.50)	50-1.95 (1.98-1.95)
No. reflections	236578	103533
Unique reflections	17636	8635
R_{merge}	0.091 (0.582)	0.135 (0.985)
I/σ (I)	32.5 (5.1)	15.4 (2.7)
Completeness (%)	97.6 (100)	99.7 (100)
Redundancy	13.4 (14.1)	12.0 (10.8)
Wilson B-factor ($Å^2$)	15.62	10.66
Refinement		
R_{work}/R_{free}	0.2461/0.2674	
No. atoms		
Protein	804	
Ligands	6	
Water	101	
Average B factor($Å^2$)	25.89	
r. m. s. deviations		
Bond length (Å)	0.006	
Bond angles (°)	0.76	
Ramachandran		
Favored (%)	100.00	
Allowed (%)	0.00	
Outliers (%)	0.00	

Statistics for the highest-resolution shell are shown in parentheses.

Supplementary Table S2

Data collection and refinement statistics of SIKE1 CC2-STRN3 complex

Data collection	
Wavelength (Å)	0.97853
Space group	$P\,2_1\,2_1\,2_1$
Cell dimensions	
a, b, c (Å)	39.28, 42.38, 91.23
α, β, γ (°)	90, 90, 90
Resolution (Å)	50-1.75 (1.81-1.75)
No. reflections	201896
Unique reflections	16205
R_{merge}	0.067 (1.245)
I/σ (I)	28.0 (2.0)
Completeness (%)	99.8 (99.4)
Redundancy	12.5 (11.4)
Wilson B-factor (Å^2)	17.05
**Refinement**	
R_{work}/R_{free}	0.1866/0.2136
No. atoms	
Protein	1053
Water	122
Average B factor(Å^2)	28.71
r. m. s. deviations	
Bond length (Å)	0.007
Bond angles (°)	0.79
Ramachandran	
Favored (%)	98.21
Allowed (%)	1.79
Outliers (%)	0.00

Statistics for the highest-resolution shell are shown in parentheses.

Supplementary Table S3

Data collection and refinement statistics of SIKE1 CC1-SLMAP CC1 complex

Data collection	
Wavelength (Å)	0.97853
Space group	$P\ 3_2\ 2\ 1$
Cell dimensions	
a, b, c (Å)	66.77, 66.77, 61.35
α, β, γ (°)	90, 90, 120
Resolution (Å)	50.00-2.30 (2.38-2.30)
No. reflections	138001
Unique reflections	7336
R_{merge}	0.094 (0.821)
I/σ (I)	21.6 (5.2)
Completeness (%)	100 (100)
Redundancy	18.8 (19.0)
Wilson B-factor (Å^2)	34.16
**Refinement**	
R_{work}/R_{free}	0.2040/0.2380
No. atoms	
Protein	732
Ligands	6
Water	37
Average B factor(Å^2)	63.28
r. m. s. deviations	
Bond length (Å)	0.007
Bond angles (°)	0.91
Ramachandran	
Favored (%)	100.00
Allowed (%)	0.00
Outliers (%)	0.00

Statistics for the highest-resolution shell are shown in parentheses.
